# Synthetic, Computational, and Experimental Studies of a Class 3 Atropisomeric α‑Naphthyl Tropone

**DOI:** 10.1021/acs.joc.5c00992

**Published:** 2025-08-05

**Authors:** Nana B. Agyemang, Jordan Nafie, Mark R. Biscoe, Ryan P. Murelli

**Affiliations:** † PhD Program in Chemistry, 14772 The Graduate Center of the City University of New York, New York, New York 10016, United States; ‡ Department of Chemistry and Biochemistry, Brooklyn College, 14770 The City University of New York, Brooklyn, New York 11210, United States; § Department of Chemistry and Biochemistry, The City College of New York, The City University of New York, New York, New York 10013, United States; ∥ Biotools, Inc., 17546 Bee Line Highway, Jupiter, Florida 33478, United States; ⊥ PhD Program in Biochemistry, The Graduate Center of the City University of New York, New York, New York 10016, United States

## Abstract

Atropisomerism is a stereochemical phenomenon that results from high configurational stability of chiral axes and is an important structural element in many functional molecules. Thus, understanding how different structural features can influence the stability of chiral axes can be an important consideration for molecular design. Recent studies have demonstrated that certain tropone-based chiral axes are significantly more stable than those of related benzenoids, likely a result of their relatively smaller external bond angles. The following manuscript explores this phenomenon through computational and experimental studies on a simple, class 3 atropisomeric α-naphthyl-substituted tropone (ΔG^‡^ ∼ 32 kcal/mol). Conditions were established for an atropselective Suzuki cross-coupling that enabled its asymmetric synthesis, and the absolute configuration of the compound was determined using vibrational circular dichroism. Based on computational modeling, we found that the α-naphthyl-substituted tropone has a lower rotational energy barrier than the phenol analog, which appears to arise from the puckering ability of the tropone. Despite the lower rotational energy barrier, the α-naphthyl-substituted tropone still displays high configurational stability. Thus, this new atropisomeric scaffold has the potential for incorporation into drug design and should support the development of new classes of ligands and catalysts for use in asymmetric synthesis.

## Graphical abstract

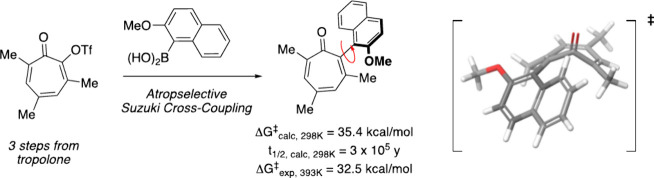

## Introduction

Atropisomerism is a stereochemical feature that arises from the high configurational stability of chiral axes, and is an important consideration in the synthesis of chiral ligands,^1^ molecular devices,^2^ and drug candidates.^3^ As such, understanding how specific molecular features influence the configurational stability of chiral axes,^4^ and synthetic methods for their construction,^5^ is of high interest. For a time, the term atropisomer was limited to axially chiral molecules with a rotational barrier energy of 22 kcal/mol or higher,^6^ as this configurational stability is generally high enough to permit isolation of individual atropisomers in optically pure form at room temperature. With recent interest in atropisomerism in drug development, a popular 3-tiered system has emerged.^7^ Within this tiering system, class 3 atropisomers are those with rotational energy barriers above 28 kcal/mol and are considered safe to develop as single atropisomer drugs. However, outside of drug development, this threshold could be higher or lower depending upon the desired application.

The Murelli lab, in collaboration with the Miller lab^8^ and subsequently Gallicchio lab,^9^ recently made the revelation that certain tropone-based chiral axes have much higher configurational stability than those of analogous benzenoid systems (e.g., Figure 1A). This phenomenon, which we suspect results from the smaller external bond angles of tropones, has significant implications with regards to the development and utility of tropone-based functional molecules and material.^10^ Very little is known about the configurational stability of tropone-based axes, which aside from our studies, is limited to studies of the axially chiral natural product colchicine and its derivatives,^11^ and axially chiral bis-tropone **3** (Figure 1B).^12^ This latter example was quite curious to us given the high rotational barriers previously observed for tropolone **2**. On the surface, **3** appears much more sterically congested than **2**, yet is ca. 10 kcal/mol less stable. While the planarity of the phenyl group is certainly involved, there is also a large lack of steric occlusion of the adjacent carbonyl group. For example, the inversion energy of the quinone-based analog of gossypol (**4**), gossypolone (**5**), is roughly 30 kcal/mol lower in energy than gossypol (Figure 1C).^13^ This raises an important fundamental question related to the stability of chiral axes that are α- to the tropone carbonyl, and whether molecules with axial chirality at this position could have class 3 atropisomeric configurational stability. Thus, we set out to test whether we could construct and study a class 3 atropisomeric troponoid with α-axial chirality.

**Figure 1:**
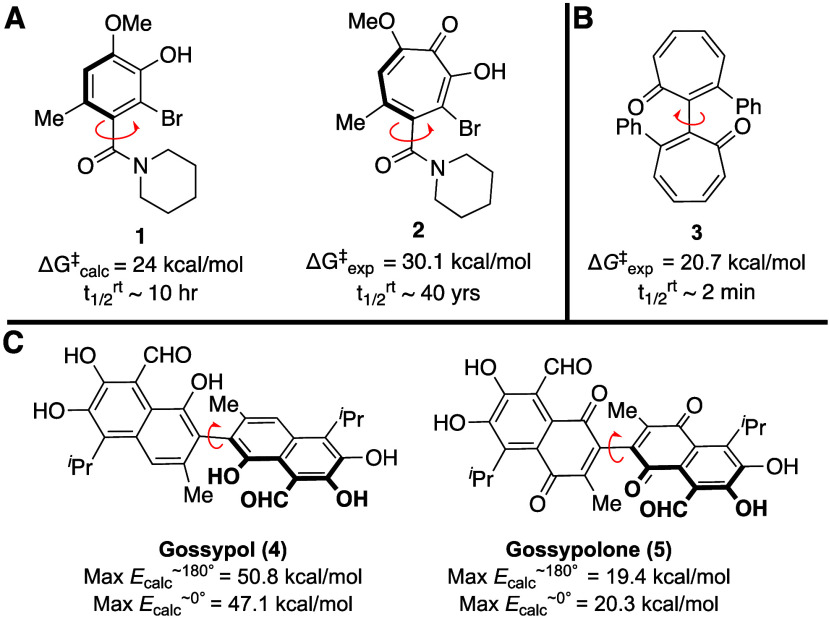
Examples of axially chiral compounds and their rotational barrier measurements.

## Results and Discussion

Our studies began with the modeling of **6a**, a simple tropone-naphthyl molecule we suspected might be atropisomeric (Figure 2A). The choice of a trimethylated troponoid portion was made based on both synthetic considerations^14^ and because methyl groups are a common benchmark for steric interactions. The methoxynaphthyl ring was similarly viewed as a good benchmark given the common usage of its corresponding boronic acid in asymmetric cross-coupling reactions.^15^ For comparison to 6-membered systems, we modeled the structurally analogous phenol (**7**) and 4-pyrone (**8**). Following geometry optimization, structures underwent a relaxed coordinate scan, starting from a geometry near the optimized structure (80° for **6a** and **8**, 60° for **7**), and modifying iteratively by 5° up to 420° (i.e., 60° past 360°) (Figure 2B–D). Transition states were then found from the local maxima through the use of the transition state search feature in Jaguar (Schrödinger Suite), and confirmed through an intrinsic reaction coordinate scan with the same software. These energy values, along with ground-state optimized structures, were used to determine the rotational barriers in Figure 2A.

**Figure 2:**
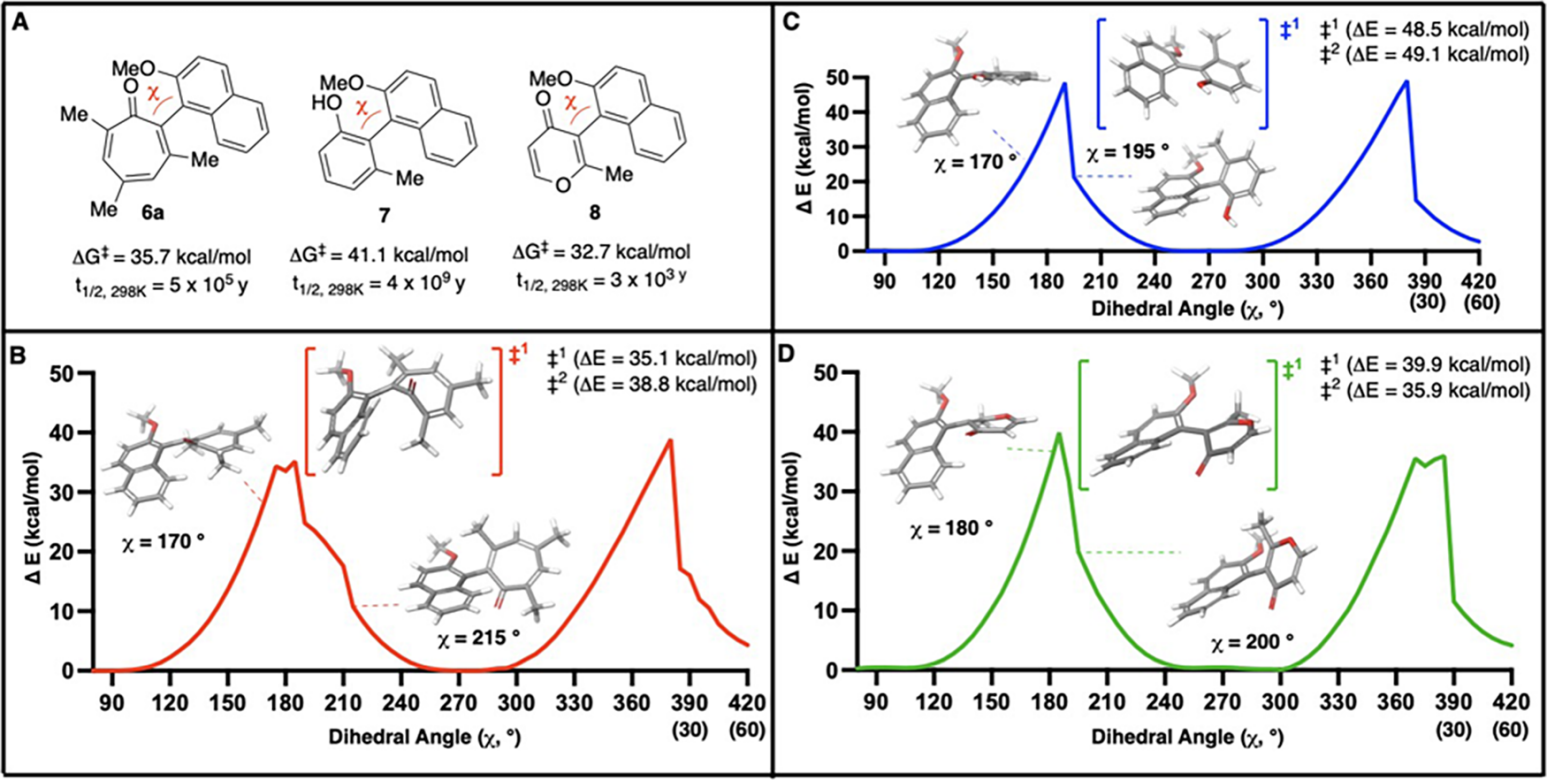
Rotational barrier measurements of select biaryls. (A) Computationally determined rotational barriers and half-lives to enantiomerization of selected biaryls. (B–D) Results from relaxed coordinate scan over one of the transition states, illustrating the dynamics of **6a** (B), **7** (C) and **8** (D) as the biaryls undergo enantiomerization. Transition state structures shown are from refined transition state search, and not the relaxed coordinate scan. All computational studies were carried out with Jaguar (Schrödinger Suite) in the gas phase at 298.15 K at B3LYP-D3/6-31G+** theory and level.

The calculated rotational barrier for **6a** suggested that the molecule would indeed be class 3 atropisomeric (Δ*G* ^‡^ = 35.7 kcal/mol), though its axial rotation was predicted to be 5 kcal/mol lower in energy than its phenol analog **7** (Δ*G* ^‡^ = 41.1 kcal/mol), and closer to that of pyrone **8** (ΔG^‡^ = 32.7 kcal/mol). Closer examimation of the structures generated through the iterative scan and transition state searches reveal some differences in how **6a** inverts its stereochemistry as compared to **7** and **8**. Specifically, as it approaches its transition states, **6a** generates a significant amount of puckering at its carbonyl bond, which aids in the rotation past the methyl group to generate a butterfly structure at its transition state (Figure 2B). The carbonyl is then able to pass the plane to complete the stereoinversion. Though **8** also puckers at its carbonyl bond, at both inversion points, it is the carbonyl that first passes the plane, followed by the methyl group (Figure 2D). **7** is more rigid than **6a** and **8**, and in the iterative dihedral scan, both groups pass the axis simultaneously. However, in the refined transition states, it becomes evident that **7** also undergoes enantiomerization in a 2-step process. At the first transition state, the methyl first passes the axis (as shown, Figure 2C), and in the second transition state it is the methoxy group that passes the axis first. Thus, in the case of **7**, it is the naphthyl ring that appears to dictate the order of interconversion, with the methoxy group representing the lower barrier.

To gain insight into the role of tropone puckering on local steric environment, the distances between *ortho* substituents at the ground and transition states were more closely evaluated (Figure 3). At their nearly perpendicular ground-states, the distance between these appendages was closest for **6a** (average = 3.67 Å) in comparison to **7** (average = 3.77 Å) and **8** (average = 3.80 Å). This can be attributed to the smaller external bond angles of tropone (118° and 112°) relative to those of pyrone and phenol (∼120°). However, this trend changes at their respective transition states, as the puckering of the tropone extends the external bond angles of **6a** (average = 125°) leading to *ortho* appendages that are further apart for **6a** (average = 2.87 Å) than for **7** (average = 2.65 Å) or pyrone **8** (2.72 Å). *We had previously proposed that tropone-enhanced configurational stability was due to the smaller external bond angles leading to closer appendages*,^7,8^ *but the extent of puckering observed in these studies provide an important caveat to this prior proposal*.

**Figure 3:**
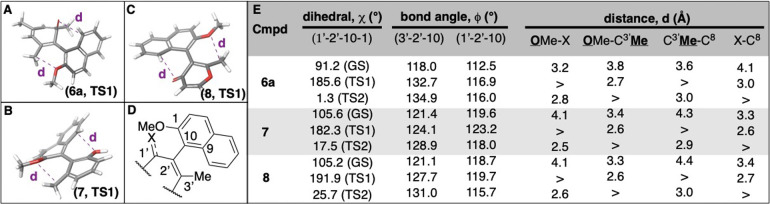
Structural features of compounds **6a**, **7**, and **8** at energetic maxima and minima. Compounds shown are the first transition state structures of **6a** (A), **7** (B) and **8** (C). (D) Representative structure **6a** and **8** (wherein 1’ is carbonyl carbon and X = O) and **7** (wherein 1’ is secondary alcohol carbon, and X = OH) with numbers to guide interpretation of Figure 3E. (E) Table of key steric-related bond angles (ϕ) and distances (d) for **6a**, **7**, and **8** at different dihedral angles. GS = Ground state. TS = Transition state. > represents large and irrelevant distances, as these appendages are on opposite ends of plane in these poses.

Given the extreme degree of puckering in the transition state, we became curious about what impact this could have on the aromaticity of the tropone. On the surface, such puckering could break aromaticity, which would be energetically unfavorable. However, many of these intermediates resemble the boat-type structure observed in homoaromatic cycloheptatriene,^16^ suggesting that the puckering may not result in a substantial loss of aromaticity. Thus, to glean insight into this possibility, we evaluated nucleus independent chemical shifts (NICS) of several of the conformations. NICS is a strategy for providing quantitative readouts of relative magnetic aromaticity by way of NMR shielding constant computations with a ‘dummy atom’ placed within the aromatic ring system (see green dots in Figure 4A).^17^ A widely used variant is NICS(1)_*zz*_, where one evaluates the zz tensor of a dummy atom 1 Å above the aromatic system,^18^ but evaluation of several points can be advantageous. In our case, the NICS(1.6)_*zz*_ or NICS(−1.6)_*zz*_ values – used to look 1.6 Å above and below the centroid – were generally higher and more consistent than those 1 Å above and below (Figure 4B). At the energy maxima (185° and 385°), which had significant boat-like conformations (for example, 185° in Figure 4A), lower NICS values were observed compared to the planar tropone in the ground-state (Figure 4B). However, some of the energetic intermediates puckered at the carbonyl without forming a boat (for example, see χ = 205° in Figure 4), and had comparable or higher NICS values relative to the planarized troponoids. This could be the result of varying homoaromaticity-enabling overlap, and indeed at χ = 205° the distance between the C2–C7 bonds is much closer than in the boat-type forms at the energy maxima. These studies suggest that the puckering does not completely destroy aromaticity, and may enhance it in certain instances. This could also help explain the shouldering observed in the transition from 190° to 210° observed in Figure 2B.

**Figure 4:**
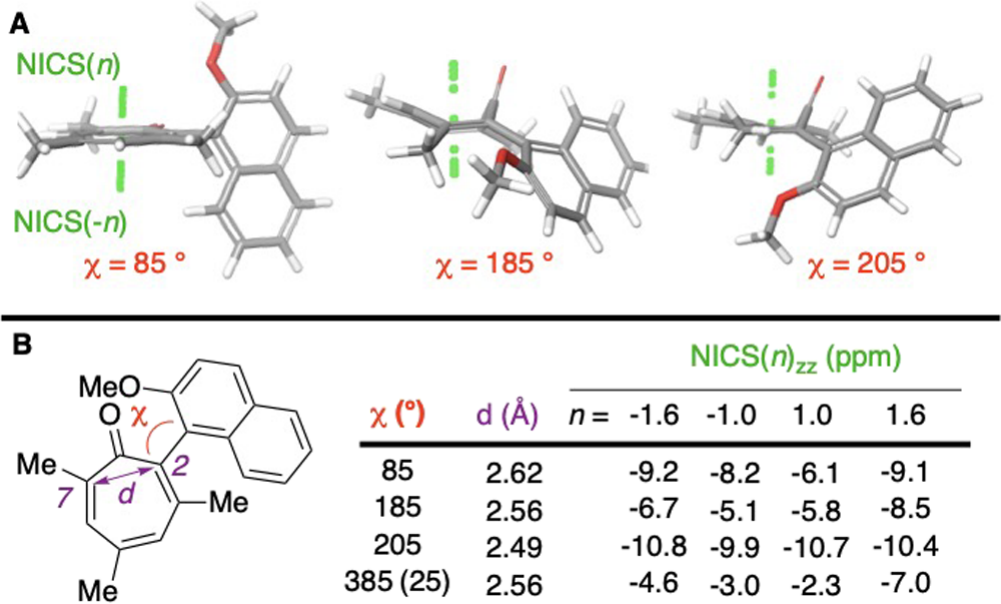
Aromaticity as determined by nucleus independent chemical shift (NICS) values. (A) Images of **6a** at key rotations and the various ‘dummy atoms’ in green. (B) NICS­(*n*) values above and below centroids at minimum (85°), intermediate conformation (205°) and two maxima (185° and 25°/ 385°). NMR shielding constants calculated at B3PW91/6–311G+** theory and level with structures obtained from aforementioned relaxed coordinate scans.

We next set out to synthesize **6a**, which was accomplished through coupling of **12** and boronic acid **13a** (Figure A). The synthesis began through an established 2-step trimethylation sequence to convert tropolone **9** into **11**,^14,19^ which was subsequently converted into the triflate **12** in high yields.^20^ Triflate **12** was then coupled to boronic acid **13a** employing standard Suzuki cross-coupling conditions to generate **6a**. These coupling conditions allowed for the synthesis of several other biaryl tropolones from **12** in moderate to excellent isolated yields (Figure 5B).

**Figure 5:**
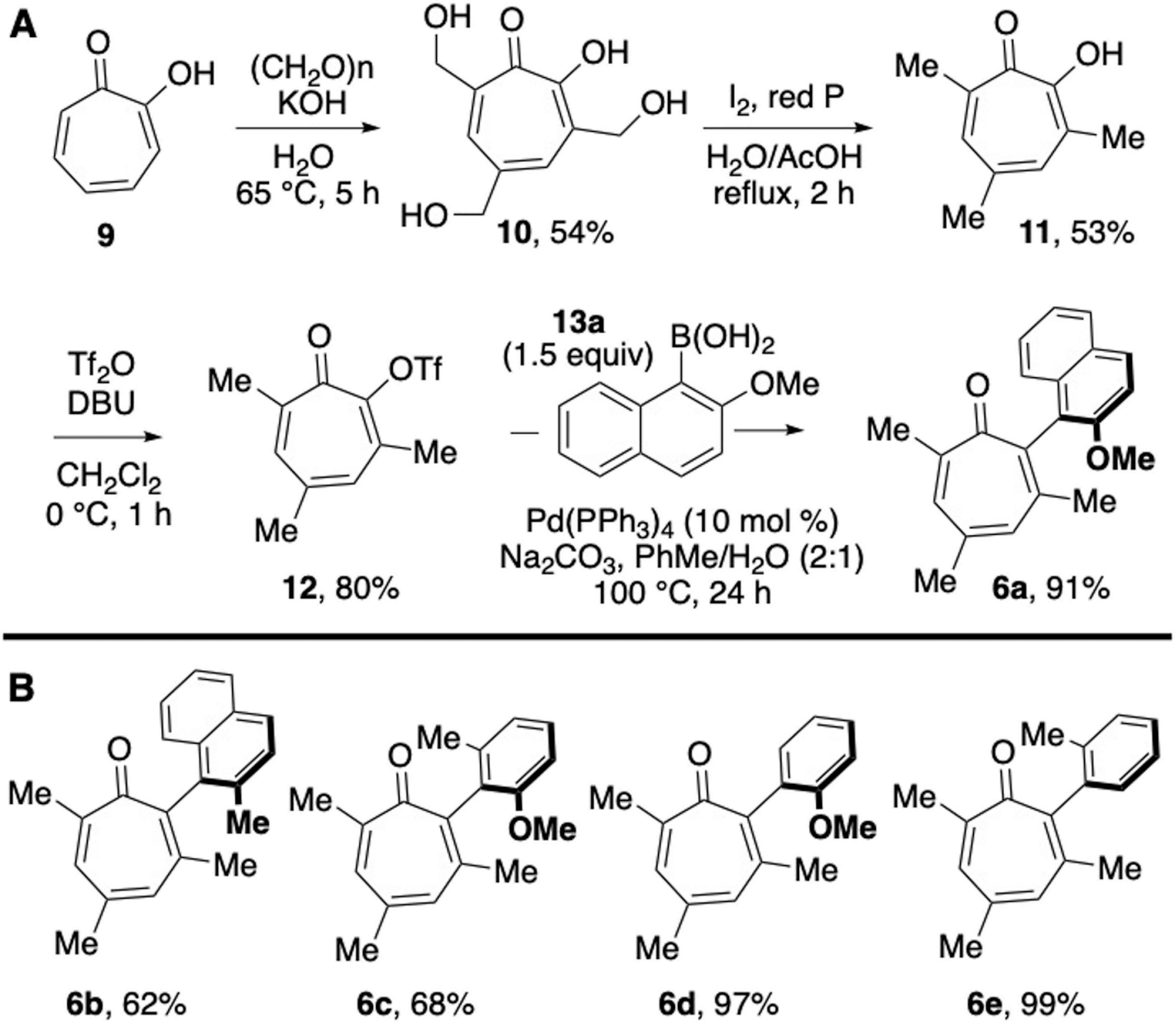
Synthesis of α-arylated tropolones. (A) Synthesis of **6a** from tropolone **9**. (B) Additional cross-coupling products prepared from **14** and the respective aryl boronic acids.

Having established coupling partners that were efficient for the racemic synthesis, attention was directed toward the identification of chiral ligands capable of inducing atropselectivity. We thus screened a variety of ligands that have been employed in transition metal-catalyzed atropselective Suzuki reactions (Figure 6 and the ). Many of these, including ferrocene-based ligand **L1** ^21^ and KenPhos (**L2**),^22^ provided little enantioselectivity. The class that provided the most promising results was benzooxaphosphole ligands (e.g., **L3**–**L8**), with **L3** providing a 90% yield and 60% enantiomeric excess (ee), or an enantiomeric ratio (er) of 80:20. Vibrational circular dichroism was used to determine absolute stereochemistry of the major enantiomer, aS, with nearly 100% confidence. Modest structural permutations to **L3**, such as changes to the substituent on the oxaphosphole backbone (**L3** vs **L4**), or to the diether (**L3** vs **L5**), had negligible effects on yields and enantioselectivities. However, more significant changes, such as removal of the group on the oxaphosphole backbone (**L5** vs **L6**), or subtractions or additions from the aryl substituent (**L7** or **L8** vs **L3**), led to significantly lower yields and selectivities.

**Figure 6:**
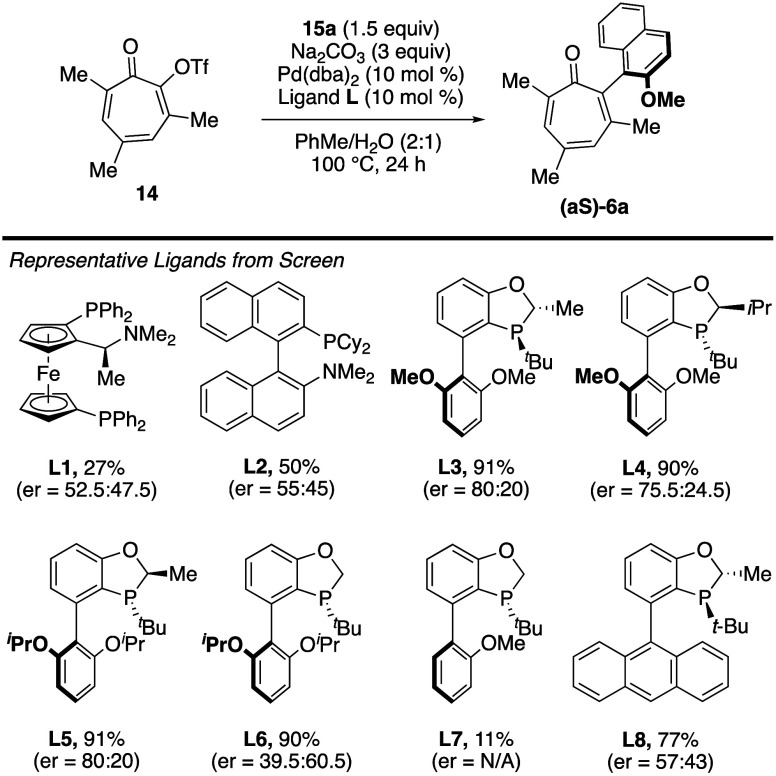
Representative results from ligand screen. Enantiomeric ratios (er) represent ratio of aS:aR; yields determined using gas chromatography with dodecane as an internal standard.

Having identified a catalyst system that provided moderate enantioselectivities, a series of systematic studies were carried out to gauge the influence of solvent, temperature, and base additives.^23^ In these studies, we opted to employ a preformed palladium precatalyst as our Pd/L source as such precatalysts have been shown to undergo facile activation to the catalytically active LPd(0) complex at low temperatures (see **G4Pd-L3**, Figure 7).^24^ Optimized conditions led to formation of **6a** in 69% yield and with a 90:10 enantiomeric ratio on gram-scale (Figure 7). Surprisingly, when naphthylboronic acid **15a** was switched to a methyl-substituted analogue, we did not observe formation of cross-coupling product **6b** using **G4Pd-L3** as precatalyst. However, by switching to the ligand **L5**-based precatalyst, yields and enantioselectivities were restored. This may suggest that **L5** is a more general ligand for the atropisomeric cross-coupling of troponoids. Truncation of the naphthyl ring to a benzenoid resulted in comparable yields to the naphthyl systems, but with diminished enantioselectivities (see **6c**). Finally, non-atropisomeric substrates **6d** and **6e** were also generated in excellent yields using the reaction conditions.

**Figure 7:**
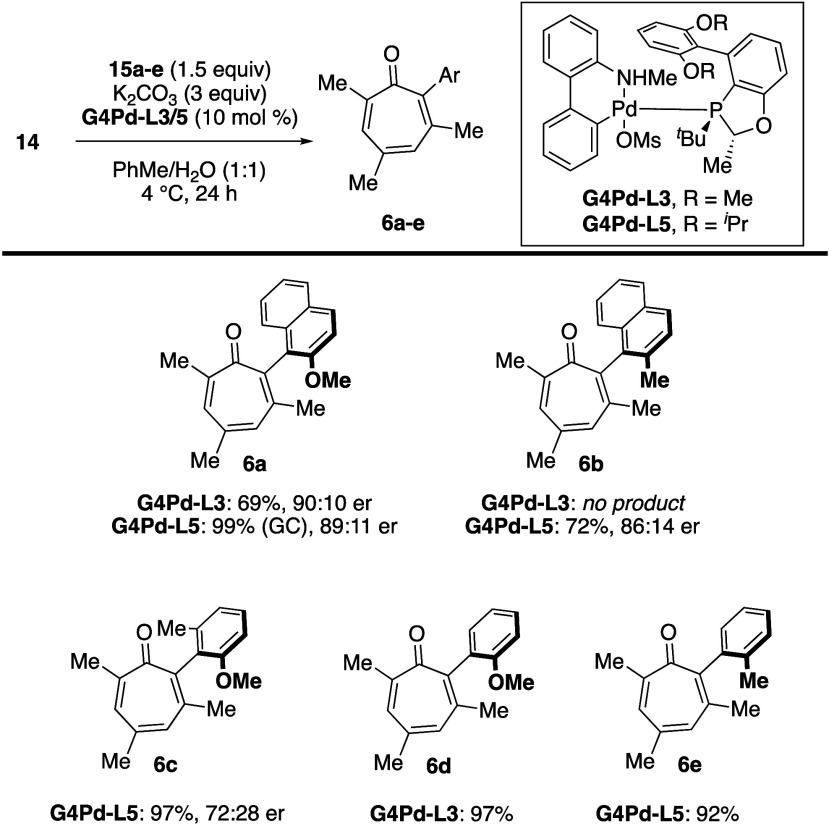
Optimized conditions and substrate scope with isolated yields.

In order to obtain experimental rotational energy barriers of **6a** and **6b**, racemization of the compounds was monitored by chiral HPLC at elevated temperatures. Clean and significant amounts of racemization (78% ee to 4% ee) of **6a** in *m*-xylenes was observed over 2 days at 120 °C (blue line, Figure 8A). From these studies, we determined a free energy barrier (Δ*G* ^‡^) of 32.5 kcal/mol, which was slightly lower than 35.7 kcal/mol computed in the gas phase at room temperature (Figure 2A). Racemization of **6b** was much more sluggish, and as a result, the free energy barrier had to be extrapolated from a modest decrease in enantioselectivity (74% ee to 63% ee) over 1 week in a solution in diphenyl ether at 175 °C (red line, Figure 8A, Δ*G* ^‡^ = 40.6 kcal/mol). For completeness, we also computationally modeled the rotational barrier of **6b**, in analogy to studies on **6a**, **7**, and **8**. These studies revealed a comparable rotational energy barrier (Δ*G* ^‡^ = 43.1 kcal/mol).

**Figure 8:**
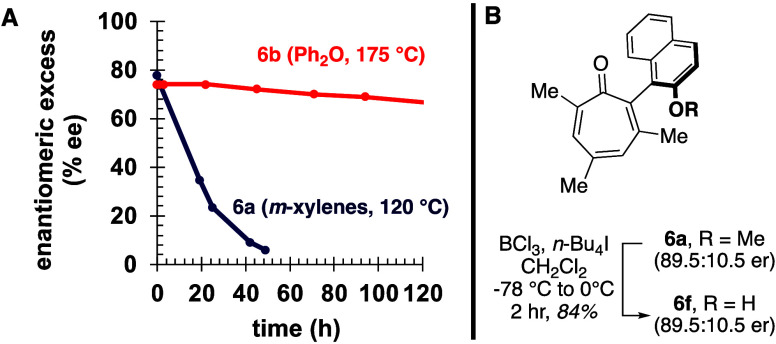
Configurational stability of (A) **6a** and **6b** to thermal racemization, and (B) **6a** to chemical modification.

The high rotational energy barriers of α-naphthyl-substituted tropones **6a** and **6b**, alongside the simple synthetic routes for their preparation, make α-naphthyl-substituted tropones and their analogues attractive candidates for use in functional molecule design. For example, there has been interest recently in the catalytic activity of troponoids,^25^ with activity that is mechanistically comparable to the much more well studied cyclopropenone derivatives.^26^ Chiral cyclopropenone catalysts have been prepared and used effectively in asymmetric reactions,^27^ and chiral troponoid catalysts based on **6a** could constitute a complementary scaffold. A logical first step for any further modification would be demethylation of **6a**. Thus, we demethylated **6a** with boron trichloride to form **6f**, demonstrating that this modification can be accomplished without adversely impacting stereochemical integrity (Figure 8B). Studies aimed at expanding upon this scaffold and evaluating its ability to catalyze enantioselective transformations are ongoing.

## Conclusions

We have found that axially chiral α-naphthyl substituted tropones have significantly lower energy barriers to racemization than analogous phenols, and exhibit racemization barriers closer to those of quinones. This lower energy barrier is likely the result of carbonyl puckering, which eases steric strain at its transition states. Despite the lower barrier to racemization, simple class 3 atropisomeric analogs can be readily isolated and manipulated synthetically. To facilitate studies of axially chiral α-naphthyl substituted tropones, atropselective Suzuki cross-coupling conditions have been developed that employ commercially available chiral benzooxaphosphole ligands, and provide good yields and enantioselectivities. Current efforts are ongoing to explore the coordinative ability of these scaffolds and their ability to be used as asymmetric catalysts or as ligands in transition metal-catalyzed transformations.

## Experimental Section

### General Information

All starting materials and reagents were purchased from commercially available sources and used without further purification. NMR spectra were recorded on a Bruker 300 MHz instrument. ^1^H NMR experiments are reported in δ units, parts per million (ppm), and were measured relative to the signals for residual chloroform (7.26 ppm), DMSO (2.50 ppm), methylene chloride (5.32 ppm) or acetone (2.05 ppm) and reported as follows: chemical shift, multiplicity (s = singlet, bs = broad singlet, d = doublet, t = triplet, dd = doublet of doublet, td = triplet of doublet, q = quartet, m = multiplet), coupling constant (Hz), and integration. All ^13^C NMR spectra are reported in ppm relative to deuterochloroform (77.23 ppm) or DMSO (39.52 ppm) and were obtained with ^1^H decoupling. Infrared (IR) spectral bands are characterized as broad (br), strong (s), medium (m), and weak (w). All previously unreported compounds were additionally characterized by high resolution MS. All high-resolution MS analyses were performed on an Agilent 6520 Q-TOF instrument. All GC analyses were performed on a Shimadzu GC-2010 gas chromatograph with an FID detector using a 25 m x 0.20 mm capillary column with cross-linked methyl siloxane as the stationary phase. All GC yields were calibrated using dodecane as an internal standard. Chiral HPLC analyses were performed on a Shimadzu LC-20AB prominence liquid chromatograph. Anhydrous THF (inhibitor-free), diethyl ether, dichloromethane, and toluene were purified by passing through two packed columns of neutral alumina. Distilled water was used in Suzuki reactions which was degassed prior to use. Flash chromatography was performed using Silicylcle silica gel (ultrapure grade). Solvents used for chromatography (ACS grade) were purchased from Fisher and used as received.

### Synthesis

#### 2,4,6-Trimethyl-7-oxocyclohepta-1,3,5-trien-1-yl Trifluoromethane­sulfonate (**12**)

An oven-dried round-bottom flask was charged with 2-hydroxy-3,5,7-trimethylcyclohepta-2,4,6-trien-1-one (**11**)^13^ (1.8 g, 10.9 mmol), anhydrous DCM (100 mL), and DBU (3.32 mL, 21.9 mmol) under an inert argon atmosphere. The contents of the flask were cooled to 0 °C. Triflic anhydride (4.6 mL, 27.25 mmol) was then added dropwise to the reaction mixture while stirring under argon. After 1 h, the reaction was quenched with a 1 M HCl solution and the reaction solution was washed consecutively with water and brine. The organic solution was dried with sodium sulfate and concentrated under reduced pressure. The crude product was purified via column chromatography (DCM) to afford **12** as a yellow solid (2.55 g, 80% yield). Mp = 68–70 °C. ^1^H NMR (300 MHz, CDCl_3_) δ 7.33 (s, 1H), 6.80 (s, 1H), 2.43 (s, 3H), 2.39 (s, 3H), 2.38 (s, 3H). ^13^C­{^1^H} NMR (75 MHz, CDCl_3_) δ 176.7, 152.0, 149.8, 144.2, 139.4, 139.0, 132.7, 118.7 (q, *J* = 20.2 Hz) 27.4, 23.4, 22.0. ^19^F-NMR (282 MHz, CDCl_3_) δ −73.61. IR (Neat): 2945 (br), 1582 (m), 1507 (m), 1413 (m), 1374 (m), 1333 (s), 1242 (m),1190 (m), 1070 (m), 1011 (m), 922 (m), 873 (m), 821 (m), 760 (m), 718 (m) cm^–1^. HRMS (ESI+) *m*/*z*: Calcd for C_11_H_11_O_4_F_3_S^+^ [M + H] 297.0403; Found 297.0402.

#### G4-L3-Palladium Precatalyst (**G4Pd-L3**)

An oven-dried round-bottom flask was charged with Buchwald’s G4 palladium dimer (828.9 mg, 1.08 mmol), and (2*S*,3*S*)-3-(*tert*-butyl)-4-(2,6-dimethoxyphenyl)-2-methyl-2,3-dihydrobenzo­[*d*]­[1,3]­oxaphosphole **L3** (724.0 mg, 2.16 mmol). The reaction flask was evacuated and backfilled with argon, THF (22 mL) was added via syringe, and the contents of the flask were allowed to stir at rt for 12 h under an argon atmosphere. Solvent was removed via reduced pressure, and the resulting grayish solid was dried overnight under vacuum. **G4Pd-L3** was obtained as a light gray solid (1.5 g, 97% yield). Mp = decomposition at 112 °C. ^1^H NMR (300 MHz, CDCl_3_) δ 7.62 (t, *J* = 5.1 Hz, 1H), 7.58–7.49 (m, 1H), 7.41–7.27 (m, 4H), 7.19 (d, *J* = 8.2 Hz, 1H), 6.97 (m, 2H), 6.84 (d, *J* = 7.7 Hz, 1H), 6.64 (d, *J* = 7.7 Hz, 1H), 6.58–6.45 (m, 3H), 4.76–4.56 (dq, *J* = 6.57, 3.92 Hz, 1H), 4.27 (s, 3H), 3.71 (s, 3H), 2.72 (s, 3H), 2.50–2.36 (m, 3H), 1.25 (dd, *J* = 15.9, 6.6 Hz, 3H), 0.80 (d, *J* = 15.2 Hz, 9H). ^13^C­{^1^H} NMR (75 MHz, CDCl_3_) δ 163.9, 163.8, 157.9, 157.7, 141.7, 141.7, 141.3, 140.3, 140.3, 139.1, 138.4, 138.3, 135.3, 135.1, 132.0, 131.9, 130.3, 128.4, 128.3, 126.9, 126.8, 126.5, 126.2, 126.1, 125.7, 125.3, 121.2, 121.2, 118.5, 116.2, 115.7, 111.1, 111.1, 104.6, 103.8, 76.1, 75.7, 67.9, 56.14, 55.6, 40.0, 39.6, 39.5, 32.9, 32.6, 26.7, 26.7, 25.6, 19.7, 19.6. ^**31**^P NMR (121 MHz, CDCl_3_) δ 62.52. IR (Neat): 3164 (br), 2934 (br), 1588 (m), 1568 (m), 1471 (m), 1424 (m), 1285 (m), 1248 (m), 1144 (m), 1064 (m), 1020 (m), 922 (m), 766 (m), 740 (m) cm^–1^. HRMS (ESI+) *m*/*z*: Calcd for C_33_H_37_NO_3_PPd^+^ [M – OMs] 632.1546; Found 632.1563.

### Atropselective Suzuki Cross-Coupling for Troponoid-Benzenoid Axially Chiral Compounds

#### General Racemic Suzuki Cross-Coupling Procedure

An oven-dried round-bottom flask was charged with tropolone triflate **12** (59.25 mg, 0.2 mmol, 1 equiv), the appropriate boronic acid (0.3 mmol, 1.5 equiv) and potassium carbonate (82.9 mg, 0.6 mmol, 3 equiv). With the reaction flask under an atmosphere of argon, tetrakis­(triphenyl­phosphine)­palladium(0) (11.55 mg, 5 mol %, 0.01 mmol) was added, followed by degassed H_2_O (1.8 mL) and anhydrous toluene (1.8 mL). The contents of the flask were allowed to stir at 100 °C, in an oil bath, for 24 h. After the reaction mixture had cooled to rt, saturated aqueous ammonium chloride (4 mL) was added and the mixture extracted with ether (3 × 10 mL). The combined organic layers were dried over sodium sulfate and concentrated under reduced pressure. Pure product was obtained via column chromatography (DCM in diethyl ether, 0% to 15%).

#### General Atropselective Suzuki Cross-Coupling Procedure

An oven-dried round-bottom flask, submerged in an ice bath at 0 °C, was charged with tropolone triflate **12** (59.25 mg, 0.2 mmol, 1 equiv), the appropriate boronic acid (0.3 mmol, 1.5 equiv), the appropriate G4 palladium precatalyst (10 mol %, 0.02 mmol), and potassium carbonate (82.9 mg, 0.6 mmol, 3 equiv). The flask was evacuated and backfilled with argon three times. Degassed H_2_O (1.8 mL) and anhydrous toluene (1.8 mL) were added via syringe with the reaction flask under a positive pressure of argon. The contents of the flask were allowed to stir at 4 °C for 24 h. After 24 h, saturated aqueous ammonium chloride solution (4 mL) was added, and the resulting mixture was extracted with diethyl ether (3 × 10 mL). The combined organic layers were dried over sodium sulfate and concentrated under reduced pressure. Pure product was obtained via column chromatography (DCM in diethyl ether, 0 to 15%).

##### 2-(2-Methoxynaphthalen-1-yl)-3,5,7-trimethylcyclohepta-2,4,6-trien-1-one (**6a**)

(2-Methoxynaphthalen-1-yl)­boronic acid (60.6 mg) and G4 palladium precatalyst **G4Pd-L3** (14.3 mg) were employed using the general cross-coupling procedure. A yellowish solid was isolated (42 mg, 69% yield, 90:10 e.r. for atropselective process; 59 mg, 97% yield for racemic reaction). Mp = 104–106 °C. ^1^H NMR (300 MHz, CDCl_3_) δ 7.89–7.75 (m, 2H), 7.38–7.27 (m, 4H), 7.21 (s, 1H), 6.87 (s, 1H), 3.85 (s, 3H), 2.37 (s, 3H), 2.27 (s, 3H), 1.89 (s, 3H). ^13^C­{^1^H} NMR (75 MHz, CDCl_3_) δ 185.6, 153.4, 149.1, 145.9, 142.4, 141.2, 137.2, 135.2, 132.1, 129.4, 129.0, 128.3, 126.7, 124.4, 123.8, 123.5, 113.8, 56.7, 26.9, 25.8, 23.6. IR (Neat): 2941 (br), 2839 (br), 1563 (m), 1506 (m), 1460 (m), 1434 (m), 1371 (m), 1316 (m), 1250 (m), 1217 (m), 1064 (m), 1033 (m), 1017 (m), 869 (m), 821 (m), 753 (m) cm^–1^. Optical rotation: [α]_D_^25^ (c 0.71, CHCl_3_) = −115.6°. HRMS (ESI+) *m*/*z*: Calcd for C_21_H_20_O_2_^+^ [M + H] 305.1536; Found 305.1538.

##### 3,5,7-Trimethyl-2-(2-methylnaphthalen-1-yl)­cyclohepta-2,4,6-trien-1-one (**6b**)

(2-Methylnaphthalen-1-yl)­boronic acid (55.8 mg) and G4 palladium precatalyst **G4Pd-L5** (15.67 mg) were employed using the general cross-coupling procedure. A colorless oily wax was isolated (41.5 mg, 72% yield, 86:14 e.r. for atropselective process; 35.7 mg, 62% yield for racemic reaction). ^1^H NMR (300 MHz, CDCl_3_) δ 7.86–7.67 (m, 2H), 7.42–7.27 (m, 4H), 7.25–7.21 (m, 1H), 6.87 (s, 1H), 2.38 (d, *J* = 1.1 Hz, 3H), 2.27 (s, 3H), 2.20 (s, 3H), 1.82 (s, 3H).^13^C­{^1^H} NMR (75 MHz, CDCl_3_) δ 185.5, 149.3, 145.4, 144.6, 141.3, 137.2, 136.9, 135.1, 132.3, 132.3, 131.2, 128.8, 128.2, 127.1, 126.1, 124.7, 124.2, 26.8, 25.3, 23.4, 19.9. IR (Neat): 2974 (br), 2950 (br), 1567 (m), 1507 (m), 1460 (m), 1365 (m), 1313 (m), 1215 (m), 1193 (m), 1122 (m), 1033 (m), 989 (m), 958 (m), 858 (m), 810 (m), 769 (m), 740 (m) cm^–1^. Optical rotation: [α]_D_^25^ (c 0.58, CHCl_3_) = +86.4°. HRMS (ESI+) *m*/*z*: Calcd for C_21_H_20_O^+^ [M + H] 289.1587; Found 289.1590.

##### 2-(2-Methoxy-6-methylphenyl)-3,5,7-trimethylcyclohepta-2,4,6-trien-1-one (**6c**)

(2-Methoxy-6-methylphenyl)­boronic acid (49.7 mg) and G4 palladium precatalyst **G4Pd-L5** (15.67 mg) were employed using the general cross-coupling procedure. An off-white solid was isolated (52.5 mg, 97% yield, 72:28 e.r. for atropselective process; 36.4 mg, 68% yield for racemic reaction). Mp = 117–121 °C. ^1^H NMR (300 MHz, CDCl_3_) δ 7.24–7.10 (m, 2H), 6.95–6.68 (m, 3H), 3.69 (s, 3H), 2.32 (s, 3H), 2.26 (s, 3H), 1.99 (s, 3H), 1.93 (s, 3H). ^13^C­{^1^H} NMR (75 MHz, CDCl_3_) δ 185.2, 155.8, 148.9, 144.6, 143.6, 140.6, 137.0, 136.3, 135.2, 129.9, 127.8, 122.6, 108.4, 55.8, 26.8, 25.4, 23.5, 19.3. IR (Neat): 2918 (br), 1590 (m), 1521 (m), 1468 (m), 1454 (m), 1436 (m), 1374 (m), 1365 (m), 1252 (m), 1199 (m), 1040 (m), 1032 (m), 992 (m), 850 (m) cm^–1^. HRMS (ESI+) *m*/*z*: Calcd for C_18_H_20_O_2_^+^ [M + H] 269.1536; Found 269.1541.

##### 2-(2-Methoxyphenyl)-3,5,7-trimethylcyclohepta-2,4,6-trien-1-one (**6d**)

(2-Methoxyphenyl)­boronic acid (45.58 mg) and tetrakis­(triphenylphosphine)­palladium(0) (11.55 mg, 5 mol %, 0.01 mmol) were employed using the general racemic cross-coupling procedure. A yellow oil was isolated (49.3 mg, 97% racemic yield). ^1^H NMR (300 MHz, CD_2_Cl_2_) δ 7.37–7.29 (m, 1H), 7.17 (s, 1H), 7.06–6.97 (m, 3H), 6.83 (s, 1H), 3.77 (s, 3H), 2.34 (s, 3H), 2.30 (s, 3H), 2.04 (s, 3H). ^13^C­{^1^H} NMR (75 MHz, CDCl_3_) δ 185.7, 156.0, 149.3, 144.5, 144.2, 140.7, 136.9, 135.2, 130.3, 129.8, 128.4, 120.9, 111.1, 55.6, 26.8, 26.1, 23.5. IR (Neat): 2931 (br), 1577 (m), 1509 (m), 1479 (m), 1431 (m), 1398 (m), 1345 (m), 1230 (m), 1209 (m), 1190 (m), 1102 (m), 989 (m), 878 (m), 776 (m), 738 (m) cm^–1^. HRMS (ESI+) *m*/*z*: Calcd for C_17_H_18_O_2_^+^ [M + H] 255.1380; Found 255.1381.

##### 3,5,7-Trimethyl-2-(*o*-tolyl)­cyclohepta-2,4,6-trien-1-one (**6e**)

*o*-Tolylboronic acid (40.7 mg) and G4 palladium precatalyst (**G4Pd-L5**) (15.67 mg) were employed using the general cross-coupling procedure. An off-white solid was isolated (43.8 mg, 92% yield, 52:48 e.r. for atropselective process; 47.1 mg, 99% yield for racemic reaction). ^1^H NMR (300 MHz, CDCl_3_) δ 7.31–7.21 (m, 3H), 7.19 (dq, *J* = 1.33, 1.18 Hz, 1H), 6.95 (m, 1H), 6.83 (s, 1H), 2.36 (d, *J* = 1.2 Hz, 3H), 2.29 (s, 3H), 2.08 (s, 3H), 1.96 (s, 3H). ^13^C­{^1^H} NMR (75 MHz, CDCl_3_) δ 185.5, 149.5, 146.9, 144.1, 141.0, 140.9, 137.1, 135.2, 135.1, 130.1, 127.9, 127.1, 126.1, 26.8, 25.9, 23.5, 19.3. IR (Neat): 2948 (br), 1568 (m), 1519 (m), 1483 (m), 1455 (m),1370 (m), 1320 (m), 1239 (m), 1207 (m), 1192 (m), 1119 (m), 993 (m), 856 (m), 796 (m), 724 (m) cm^–1^. HRMS (ESI+) *m*/*z*: Calcd for C_17_H_18_O^+^ [M + H] 239.1430; Found 239.1432.

##### 2-(2-Hydroxynaphthalen-1-yl)-3,5,7-trimethylcyclohepta-2,4,6-trien-1-one (**6f**)

An oven-dried round-bottom flask was placed under an argon atmosphere was charged with 2-(2-methoxynaphthalen-1-yl)-3,5,7-trimethylcyclohepta-2,4,6-trien-1-one (**6a**) (50.0 mg, 0.164 mmol) and anhydrous *n*-Bu_4_NI (151.7 mg, 0.41 mmol). DCM (2 mL) was added, and the flask cooled to –78 °C. Boron trichloride (1 M in DCM, 0.41 mL, 0.41 mmol) was added dropwise over 20 min, and the reaction stirred at –78 °C for 5 min. The reaction warmed to 0 °C and stirred for 2 h. Ice water (3 mL) was then added and the resulting reaction mixture allowed to stir at 0 °C for 30 min. The DCM in the reaction was evaporated off under reduced pressure. Additional water (3 mL) was added, and the resulting mixture extracted with diethyl ether. The combined organic layers were washed with brine and dried over sodium sulfate. Purification via column chromatography (DCM in diethyl ether, 0 to 15%) afforded **6f** as an off-white solid (39.9 mg, 84% yield, 89:11 e.r.). Mp = 160–161 °C. ^1^H NMR (300 MHz, CDCl_3_) δ 7.88–7.69 (m, 2H), 7.63 (s, 1H), 7.35–7.26 (m, 4H), 7.22–7.15 (m, 1H), 6.92 (s, 1H), 2.35 (s, 3H), 2.42 (s, 3H), 1.87 (s, 3H). ^13^C­{^1^H} NMR (75 MHz, CDCl_3_) δ 189.2, 153.0, 147.4, 146.7, 142.0, 138.5, 137.2, 135.1, 132.4, 129.7, 129.1, 128.3, 126.5, 123.5, 123.1, 121.0, 120.0, 26.7, 25.7, 22.8. IR (Neat): 3207 (br), 2922 (m), 1538 (m), 1510 (m), 1431 (m), 1370 (m), 1344 (m), 1271 (m), 1219 (m), 1178 (m), 1143 (m), 1124 (m),1032 (m), 971 (m), 948 (m), 860 (m) cm^–1^. Optical rotation: [α]_*D*_^25^ (*c* 0.24, CHCl_3_) = +3.8°. HRMS (ESI+) *m*/*z*: Calcd for C_20_H_18_O_2_^+^ [M + H] 291.1380; Found 291.1381.

### Computational Studies

#### Rotational Barrier

Calculations were carried out at B3LYP-D3/6-31G+** level and theory using Schrödinger Suite Software. Structures **6a**, **6b**, **7**, and **8** were generated using Maestro v. 13.1.137 release 2022-1, and optimized using Jaguar to find ground state configurations. Next, Jaguar was used to perform an iterative relaxed coordinate scan of dihedral angles, varying from 80° to 420° in 5° increments. The local maxima were refined into a transition state using the transition state search feature in Jaguar, and accuracy of the transition states were confirmed by the presence of a single negative frequency and a successful intrinsic reaction coordinate scan leading toward the two ground-state axial chiral forms. Optimization of these structures provided ground-state configurations. Subtracting the energy maxima from the minima provided two activation energy barriers (*E*_A_) for each molecule, which were then used to calculate an observed Δ*G*_rot_, in kcal/mol using eqs 1, 2, and 3 sequentially:

1 $$K_{Transition}=\frac{K_{B}T}{h}e^{-E_{A}/RT}$$

2 $$K_{\mathrm{obs}}=K_{T1}+K_{T2}$$

3 $$\Delta G_{obs}=RT\;\ln\left(\frac{k_{obs}h}{k_{b}T}\right)$$

These values are reported for in Figure 3 for **6a**, **7**, and **8**, and described in text for **6b** (35.4 kcal/mol). Additional details can be found in the .

#### Nucleus Independent Chemical Shift Determination

Structures of **6a** at the energetic minima (85°), two maxima (185° and 25°/385°), and intermediate conformation (205°) were repositioned with the tropone in the XY plane (*ie*, Z = 0 at 85°) using the superposition feature in Maestro, and a planarized tropone as a standard. For puckered structures, given the puckered nature, alkenes at 2–3 and 6–7 were chosen to be superimposed/placed in XY plane. Initial dummy atoms were generated by adding a series of centroids between all 7 of the atoms in the tropone ring, and moving them to distances −1.6, −1.0, 0, 1.0, 1.6 Å in the Z direction. NMR shielding constants were then calculated in Jaguar using single point energy at the B3PW91/6-311G+** level and theory. The ZZ-specific shielding tensor was found in the output file for each dummy atom, and were multiplied by −1 to provide NICS­(n)_*zz*_ values reported in Figure 4.

##### Experimental Rotational Barrier Determination

Solutions of **6a** (in m-xylene) and **6b** (in diphenyl ether) were heated using oil bath to desired temperatures, and periodic aliquots were extracted and subjected to chiral HPLC analysis using chiralcel OJRH column with an isocratic 50:50 H_2_O/MeCN mobile phase. The rate constant for the rotation (*k*_enant_), was computed by plotting the change in% ee as a function of time, using the equation: $k_{enant}=\frac{\ln\left(\frac{1}{ee}\right)}{t}$ . This value of *k*_enant_ was substituted into the Eyring-Polyani equation: $\Delta G^{‡}=-RT\;\ln\left(\frac{k_{enant}h}{k_{b}T}\right)$ , where in the transmission coefficient, κ, is assumed to be unity. Additional details can be found in the .

## Supplementary Material







## Data Availability

The data underlying this study are available in the published article and its .

## Notes

Reaction condition screening, chiral HPLC traces, ^1^H and ^13^C NMR spectra of all newly synthesized compounds, data on experimental rotational barrier determination, computational data associated with rotational barrier and NICS measurements (PDF)
Full report on vibrational circular dichroism studies, as provided by Biotools (PDF)
Cartesian coordinates associated with modeling (ZIP)
