# Supplementary material for: Synthetic, Computational, and Experimental Studies of a Class 3 Atropisomeric α‑Naphthyl Tropone
Source: J Org Chem. 2025 Aug 5;90(32):11501–9. doi: 10.1021/acs.joc.5c00992 (PMC12362610; doi:10.1021/acs.joc.5c00992)
Supplement: Supplementary file 1 [file jo5c00992_si_001.pdf]

Supporting Information

Synthetic, Computational, and Experimental Studies of a Class 3 Atropisomeric α-Napthyl Tropone

Nana B. Agyemang,[a,b,c] Jordan Nafie,[d] Mark R. Biscoe,[a,c*] Ryan P. Murelli[a,b,e*]

[a] PhD Program in Chemistry, The Graduate Center of the City University of New York, New York, NY, USA

[b] Department of Chemistry and Biochemistry, Brooklyn College, The City University of New York, Brooklyn, NY, USA

[c] Department of Chemistry and Biochemistry, City College of New York, The City University of New York, New York, NY, USA

[d] Biotools, Inc., 17546 Bee Line Highway, Jupiter, FL, USA

[e] PhD Program in Biochemistry, The Graduate Center of the City University of New York, New York, NY, USA

Table of Contents

Screening Tables S3
Racemic reaction run S3
Ligand S4
Solvent S5
Temperature S5
HPLC Data S6
Compound **6a** racemic S6
Compound **6a** enantioenriched S7
Compound **6b** racemic S8
Compound **6b** enantioenriched S9
Compound **6c** racemic S10
Compound **6c** enantioenriched S11
Compound **6e** racemic S12
Compound **6e** enantioenriched S13
Compound **6f** racemic S14
Compound **6f** enantioenriched S15
NMR spectra S16
^{1}H-NMR of compound **11** S16
^{13}C-NMR of compound **11** S16
^{1}H-NMR of compound **12** S17
^{13}C-NMR of compound **12** S17
^{19}F-NMR of compound **12** S18
^{1}H-NMR of pre-catalyst **G4Pd-L3** S18
^{13}C-NMR of pre-catalyst **G4Pd-L3** S19
^{31}P-NMR of pre-catalyst **G4Pd-L3** S19
^{1}H-NMR of compound **6a** S20
^{13}C-NMR of compound **6a** S20
^{1}H-NMR of compound **6b** S21
^{13}C-NMR of compound **6b** S21
^{1}H-NMR of compound **6f** S22
^{13}C-NMR of compound **6f** S22
^{1}H-NMR of compound **6d** S23
^{13}C-NMR of compound **6d** S23
^{1}H-NMR of compound **6e** S24
^{13}C-NMR of compound **6e** S24
^{1}H-NMR of compound **6c** S25
^{13}C-NMR of compound **6c** S25
Racemization Studies S26
Computational Rotational Barrier Measurements S27
Nuclear Independent Chemical Shift Measurements. S29

Screening Tables

All screening reactions were performed using electrophile (1.0 equiv), nucleophile (1.5 equiv), palladium source (10 mol %), ligand (10 mol %), base (3.0 equiv), and a 2:1 ratio of toluene to water, unless stated differently. Product yields were determined using GC and dodecane as the internal standard. Enantiomeric excess was determined using chiral-phase HPLC.

Racemic reaction run:

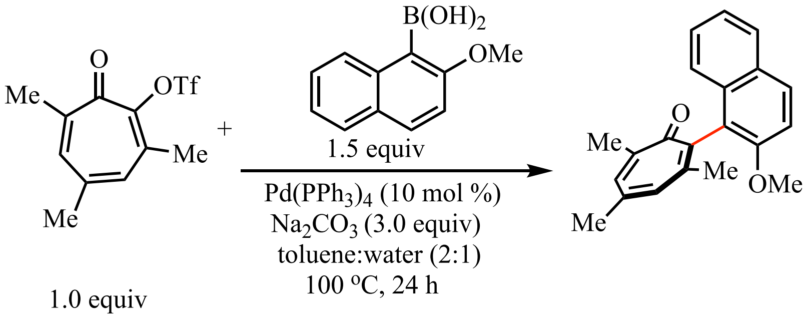

entry	Substrate	yield (%)
1	OTf	91

Scheme SI 1. Racemic reaction run

*Ligand screen: (**L1–L16** were all purchased from Strem and used as received)*

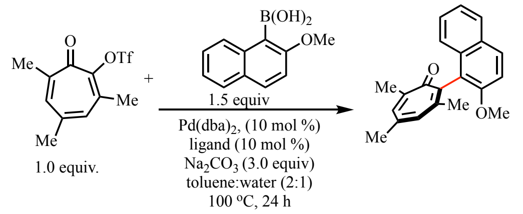

entry	ligand	yield (%)	% ee
1	**L1**	27.1	5
2	**L2**	50	10
3	**L9**	no product	—
4	**L10**	91	60

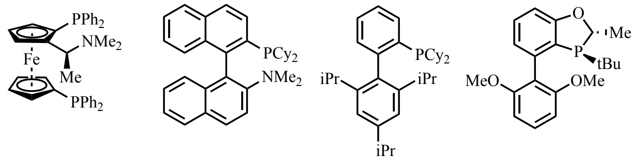

L1 **L2** **L9** **L10**

Scheme SI 2. Ligand class screening

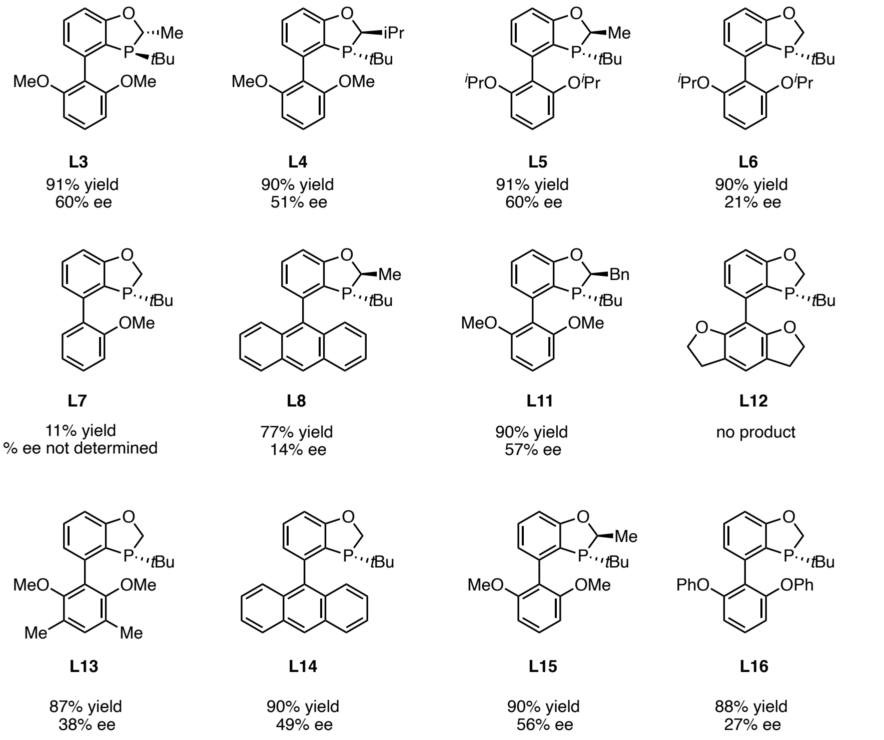

Figure SI 1. Chiral monophosphine ligands screened for atropselective Suzuki cross-coupling reactions.

Solvent screen:

entry	solvent	yield (%)	% ee
1	Toluene:Water (2:1)	44	79
2	N,N-Dimethylacetamide	47	76
3	DME:Water (2:1)	57	75
4	*t*-Butanol:Water (2:1)	55	76
5	Acetonitrile:Water (2:1)	10	58
6	1,4-Dioxane:Water (2:1)	34	74
7	THF:Water (2:1)	49	77
8	Toluene	26	61
9	Toluene:Water (1:1)	43	81
10	Toluene: Water (5:1)	39	80
11	Toluene:Water (1:3)	54	75
12	Water	no product	

Figure SI 2. Solvent and solvent ratio screening

Temperature screen:

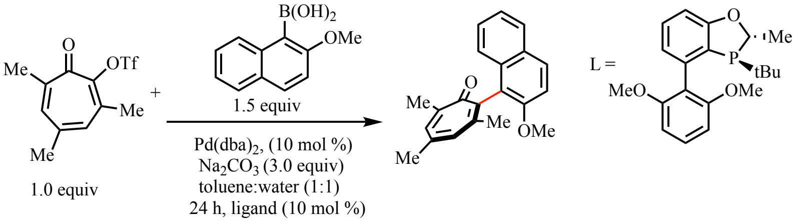

entry	temperature (ºC)	yield (%)	% ee
1	100	91	60
2	60	90	65
3	25	89	68
4	4	44	79

Scheme SI 3. Temperature screening

Chiral HPLC Data

Instrument: Shimadzu LC-20AB, with SPD-20A dual-channel UV detector

Column: Chiralcel OJRH

Mobile Phase: A: H_2O; B: Acetonitrile (50% B)

Method: 1.0 mL/min isocratic

HPLC of Racemic **6a**

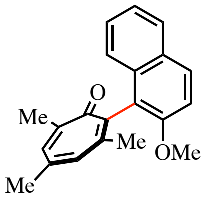

Page 1 of 1

Area % Report

Data File: C:\EZStart\Data\NBA\NBA Tropolone\NBA-II-34A-CONDITION 22.dat
Method: C:\EZStart\Methods\Anand\CC condition1 OJRH.met
Acquired: 5/19/2021 10:34:07 AM
Printed: 7/17/2023 2:49:30 PM

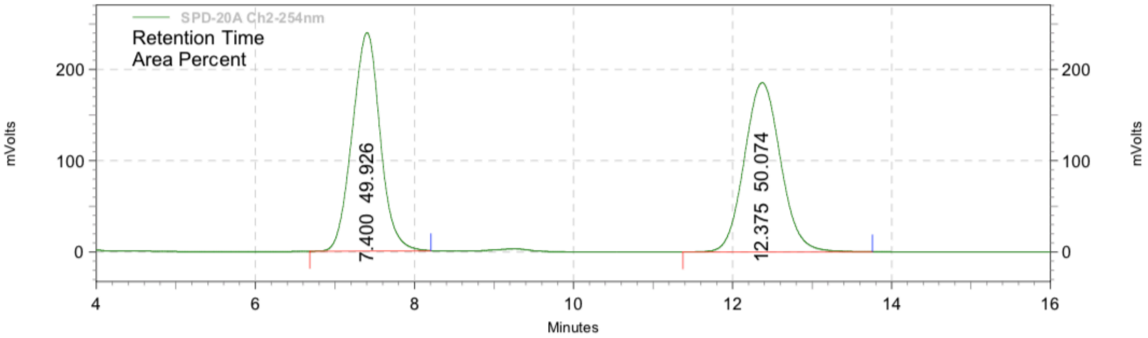

SPD-20A
Ch2-254nm
Results

Retention Time	Area	Area %	Height	Height %
7.400	5848299	49.93	239375	56.36
12.375	5865628	50.07	185353	43.64
Totals	11713927	100.00	424728	100.00

Column: Chiralcel OJRH

Mobile Phase: A: H_2O; B: Acetonitrile (50% B)

Method: 1.0 mL/min isocratic

HPLC of Enantioenriched **6a**

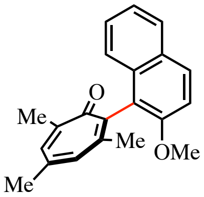

Area % Report

Data File: C:\EZStart\Data\NBA\NBA Tropolone\NBA-II-157-Xtals in Hex-1.dat
Method: C:\EZStart\Methods\Anand\CC condition1 OJRH.met
Acquired: 6/16/2023 2:57:39 PM
Printed: 7/17/2023 2:55:48 PM

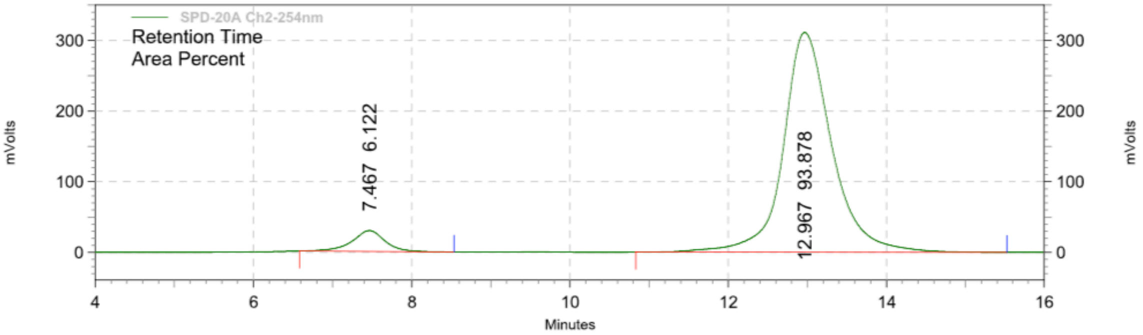

SPD-20A
Ch2-254nm
Results

Retention Time	Area	Area %	Height	Height %
7.467	865910	6.12	29570	8.68
12.967	13277432	93.88	311030	91.32
Totals	14143342	100.00	340600	100.00

Column: Chiralcel OJRH

Mobile Phase: A: H_2O; B: Acetonitrile (50% B)

Method: 1.0 mL/min isocratic

HPLC of Racemic **6b**

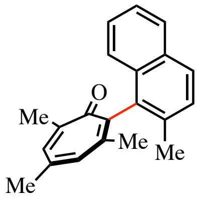

Page 1 of 1

Area % Report

Data File: C:\EZStart\Data\NBA\NBA Tropolone\NBA-II-122B-Racemic.dat
Method: C:\EZStart\Methods\NBA\General Start-up Method.met
Acquired: 11/9/2022 1:03:06 PM
Printed: 7/17/2023 3:58:00 PM

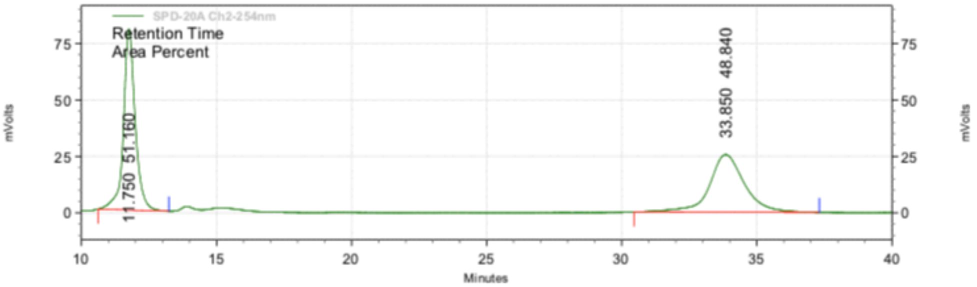

SPD-20A
Ch2-254nm
Results

Retention Time	Area	Area %	Height	Height %
11.750	2423749	51.16	80058	75.84
33.850	2313874	48.84	25507	24.16
Totals	4737623	100.00	105565	100.00

Column: Chiralcel OJRH

Mobile Phase: A: H_2O; B: Acetonitrile (50% B)

Method: 1.0 mL/min isocratic

HPLC of Enantioenriched **6b**

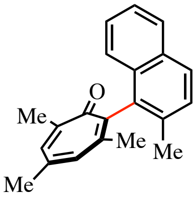

Page 1 of 1

Area % Report

Data File: C:\EZStart\Data\NBA\NBA Tropolone\NBA-III-42-Starting ee 8-fresh from solid.dat
Method: C:\EZStart\Methods\NBA\General Start-up Method.met
Acquired: 11/9/2022 4:06:57 PM
Printed: 7/17/2023 4:02:16 PM

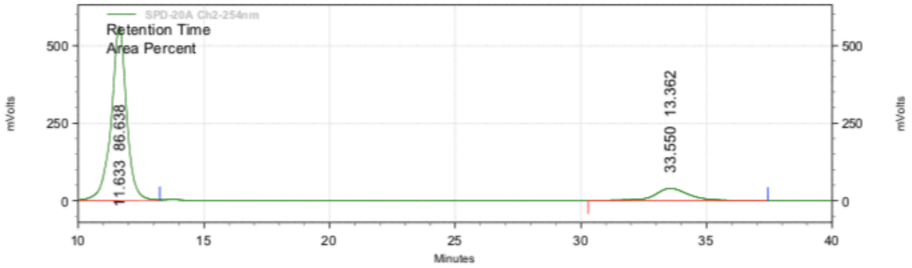

SPD-20A
Ch2-254nm
Results

Retention Time	Area	Area %	Height	Height %
11.633	23764287	86.64	560387	93.47
33.550	3665225	13.36	39119	6.53
Totals	27429512	100.00	599506	100.00

Column: Chiralcel OJRH

Mobile Phase: A: H_2O; B: Acetonitrile (30% B)

Method: 0.8 mL/min isocratic

HPLC of Racemic **6c**

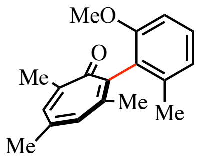

Area % Report

Data File: C:\EZStart\Data\NBA\NBA Tropolone\NBA-III-112-Racemic-4.dat
Method: C:\EZStart\Methods\NBA\General Start-up Method.met
Acquired: 5/3/2023 1:55:13 PM
Printed: 8/28/2023 2:50:46 PM

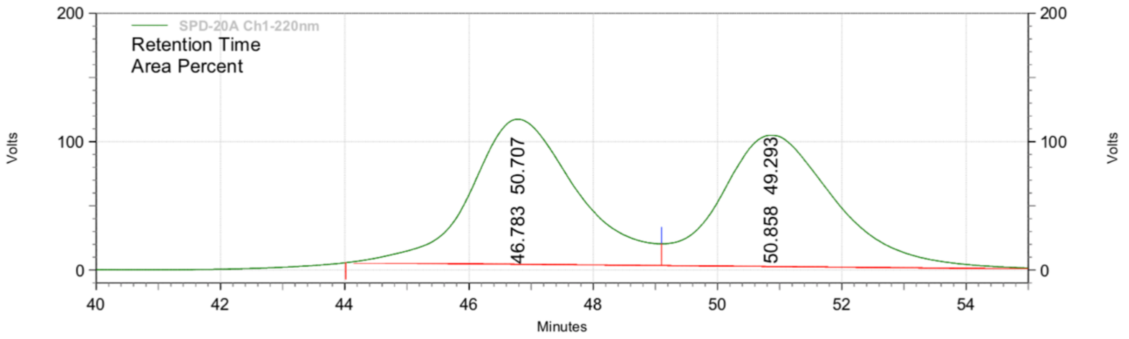

SPD-20A
Ch1-220nm
Results

Retention Time	Area	Area %	Height	Height %
46.783	13526082	50.71	112915	52.49
50.858	13149091	49.29	102217	47.51
Totals	26675173	100.00	215132	100.00

Column: Chiralcel OJRH

Mobile Phase: A: H_2O; B: Acetonitrile (30% B)

Method: 0.8 mL/min isocratic

HPLC of Enantioenriched **6c**

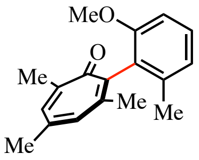

Area % Report

Data File: C:\EZStart\Data\NBA\NBA Tropolone\NBA-III-117-Chiral-1.dat
Method: C:\EZStart\Methods\NBA\General Start-up Method.met
Acquired: 5/8/2023 1:44:12 PM
Printed: 8/28/2023 2:54:35 PM

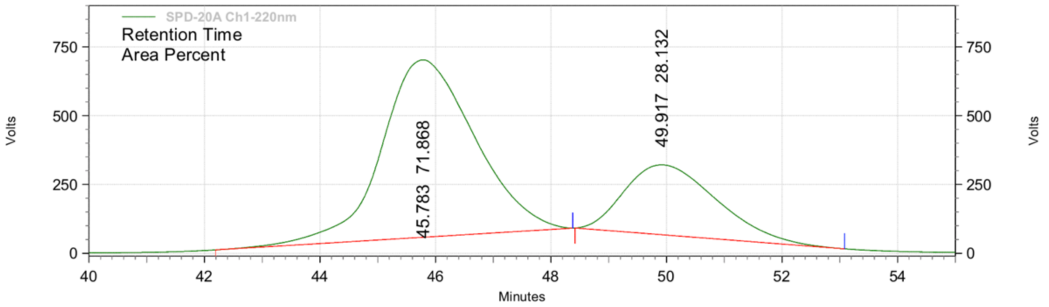

SPD-20A
Ch1-220nm
Results

Retention Time	Area	Area %	Height	Height %
45.783	72397261	71.87	644403	71.72
49.917	28339490	28.13	254105	28.28
Totals	100736751	100.00	898508	100.00

Column: Chiralcel OJRH

Mobile Phase: A: H_2O; B: Acetonitrile (70% B)

Method: 1.0 mL/min isocratic

HPLC of Racemic **6e**

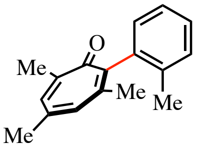

Area % Report

Data File: C:\EZStart\Data\NBA\NBA Tropolone\NBA-III-5-Racemic-2.dat
Method: C:\EZStart\Projects\Default\Method\NBA\General Start-up Method.met
Acquired: 12/16/2021 1:07:20 PM
Printed: 8/6/2023 12:53:21 PM

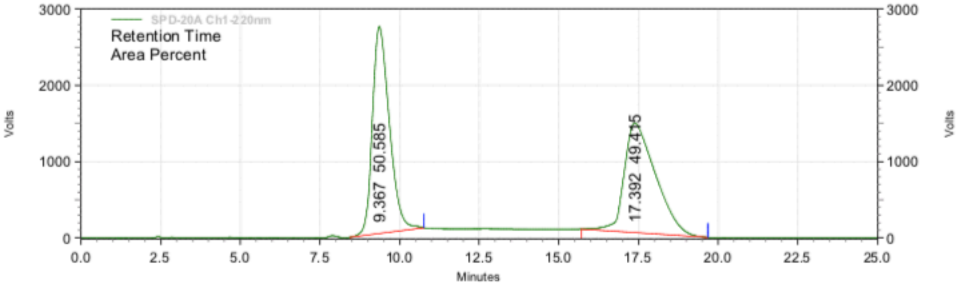

SPD-20A
Ch1-220nm
Results

Retention Time	Area	Area %	Height	Height %
9.367	100439365	50.58	2712871	65.37
17.392	98117333	49.42	1437098	34.63
Totals	198556698	100.00	4149969	100.00

Column: Chiralcel OJRH

Mobile Phase: A: H_2O; B: Acetonitrile (70% B)

Method: 1.0 mL/min isocratic

HPLC of Enantioenriched **6e**

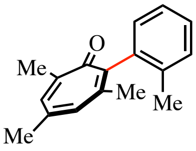

Area % Report

Data File: C:\EZStart\Data\NBA\NBA Tropolone\NBA-III-7-A-0.dat
Method: C:\EZStart\Projects\Default\Method\pej\temp 75 iso.met
Acquired: 12/23/2021 12:39:06 PM
Printed: 8/6/2023 12:59:15 PM

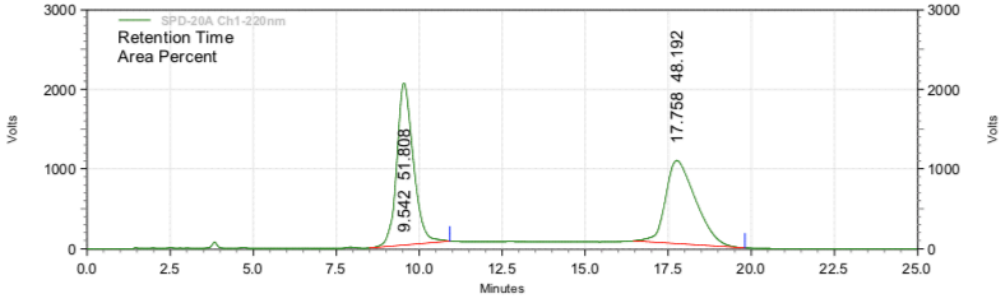

SPD-20A
Ch1-220nm
Results

Retention Time	Area	Area %	Height	Height %
9.542	70468566	51.81	2032334	66.10
17.758	65549463	48.19	1042350	33.90
Totals	136018029	100.00	3074684	100.00

Column: Chiralpak IA

Mobile Phase: A: Isopropanol; B: Hexane (50% B)

Method: 0.5 mL/min isocratic

HPLC of Racemic **6f**

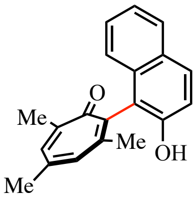

Page 1 of 1

Area % Report

Data File: C:\EZStart\Data\NBA\NBA Tropolone\NBA-III-132-Rac-15-1.dat
Method: C:\EZStart\Methods\NBA\General Start-up Method.met
Acquired: 6/27/2023 2:43:04 PM
Printed: 7/17/2023 4:12:05 PM

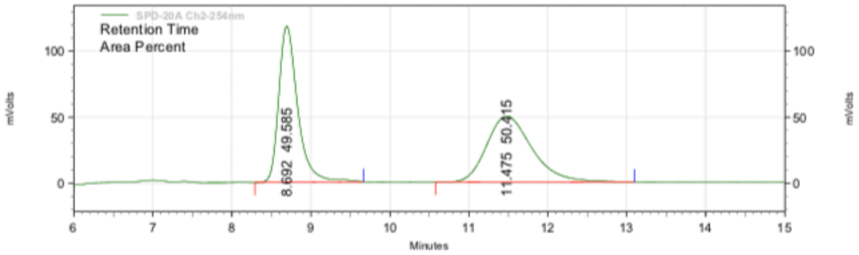

SPD-20A
Ch2-254nm
Results

Retention Time	Area	Area %	Height	Height %
8.692	1970573	49.58	118618	70.28
11.475	2003598	50.42	50154	29.72
Totals	3974171	100.00	168772	100.00

Column: Chiralpak IA

Mobile Phase: A: Isopropanol; B: Hexane (50% B)

Method: 0.5 mL/min isocratic

HPLC of Enantioenriched **6f**

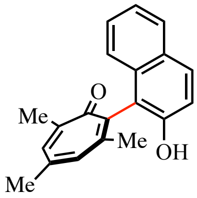

Page 1 of 1

Area % Report

Data File: C:\EZStart\Data\NBA\NBA Tropolone\NBA-III-128-ChiralRun-0.dat
Method: C:\EZStart\Methods\NBA\General Start-up Method.met
Acquired: 6/28/2023 10:16:23 AM
Printed: 7/17/2023 4:15:41 PM

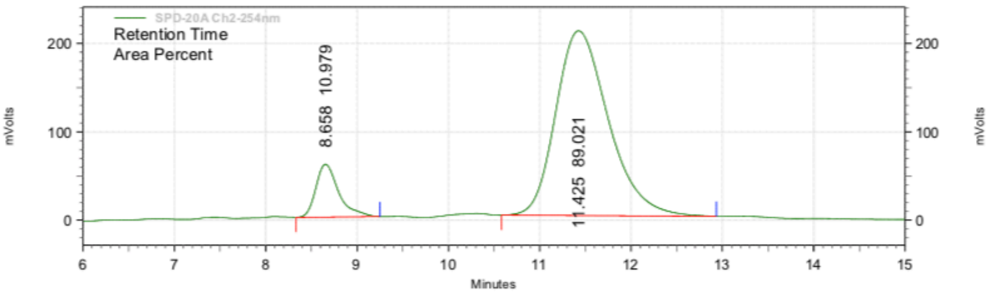

SPD-20A
Ch2-254nm
Results

Retention Time	Area	Area %	Height	Height %
8.658	1034307	10.98	59729	22.22
11.425	8386386	89.02	209100	77.78
Totals	9420693	100.00	268829	100.00

NMR spectra of synthesized compounds.

^{1}H-NMR (300 MHz) of 11

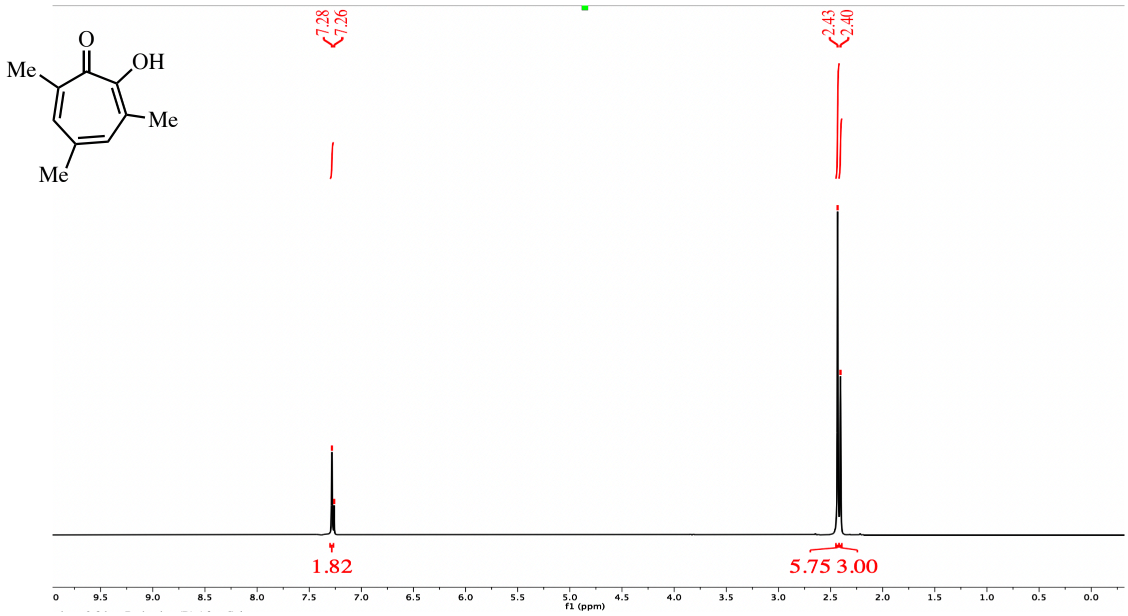

^{13}C{^{1}H}-NMR (75 MHz) of 11

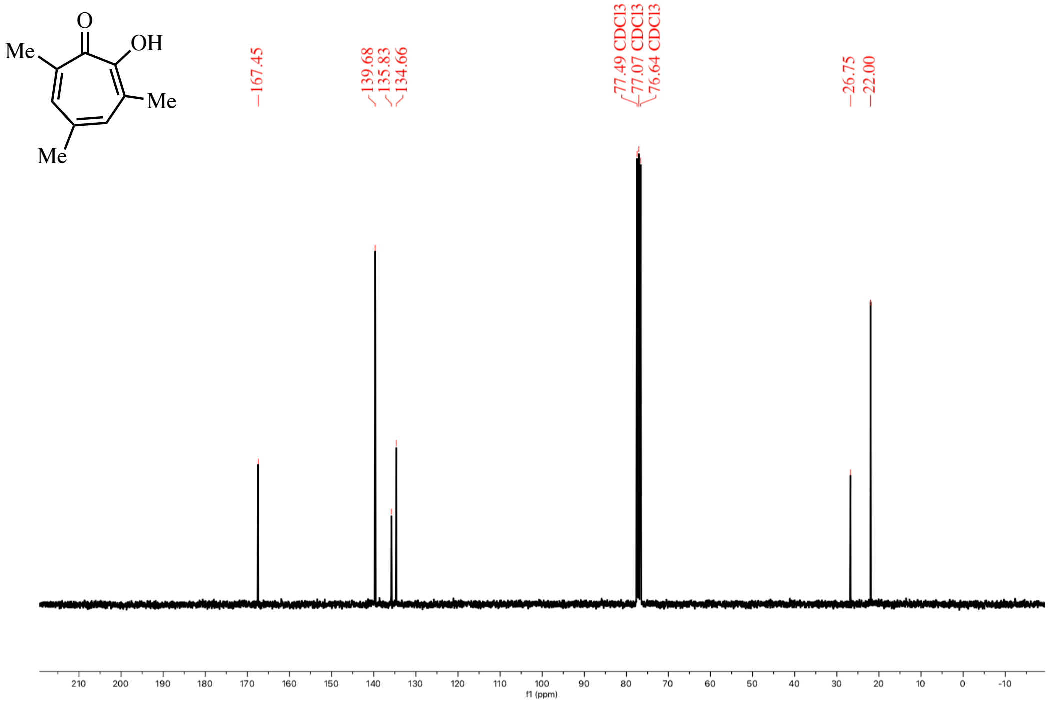

^{1}H-NMR (300 MHz) of 12

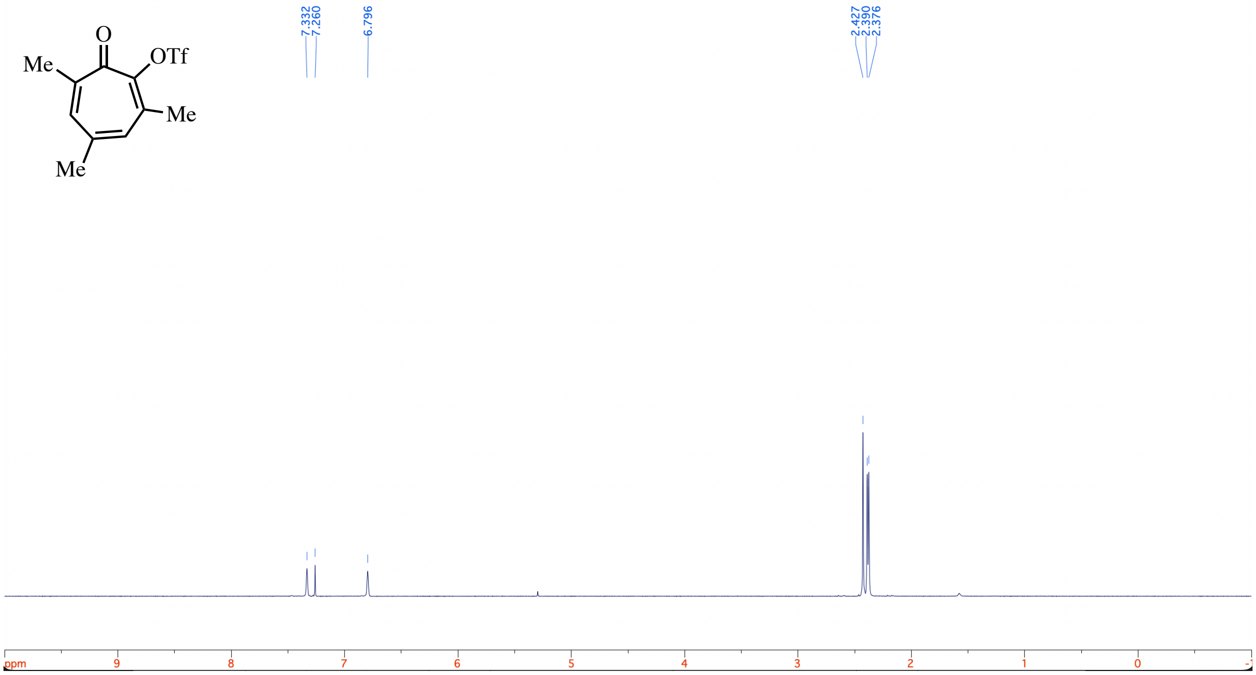

^{13}C{^{1}H}-NMR (75 MHz) of 12

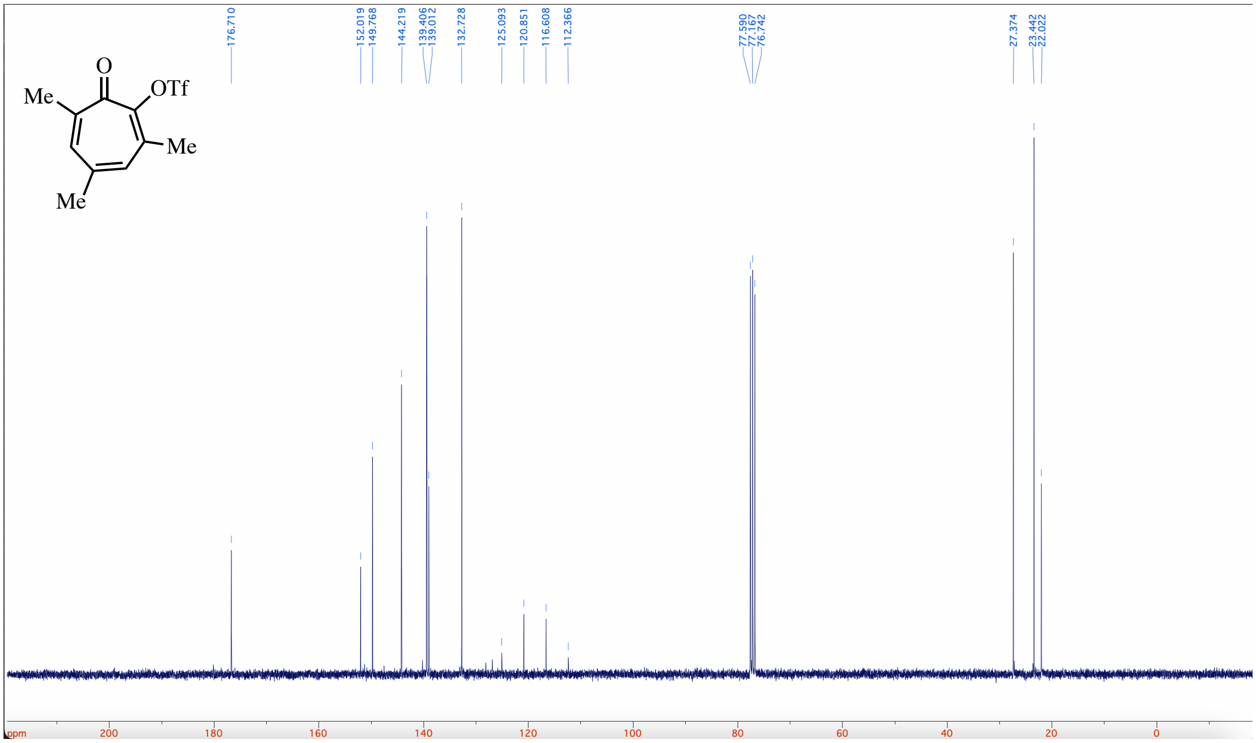

^{19}F-NMR (282 MHz) of 12

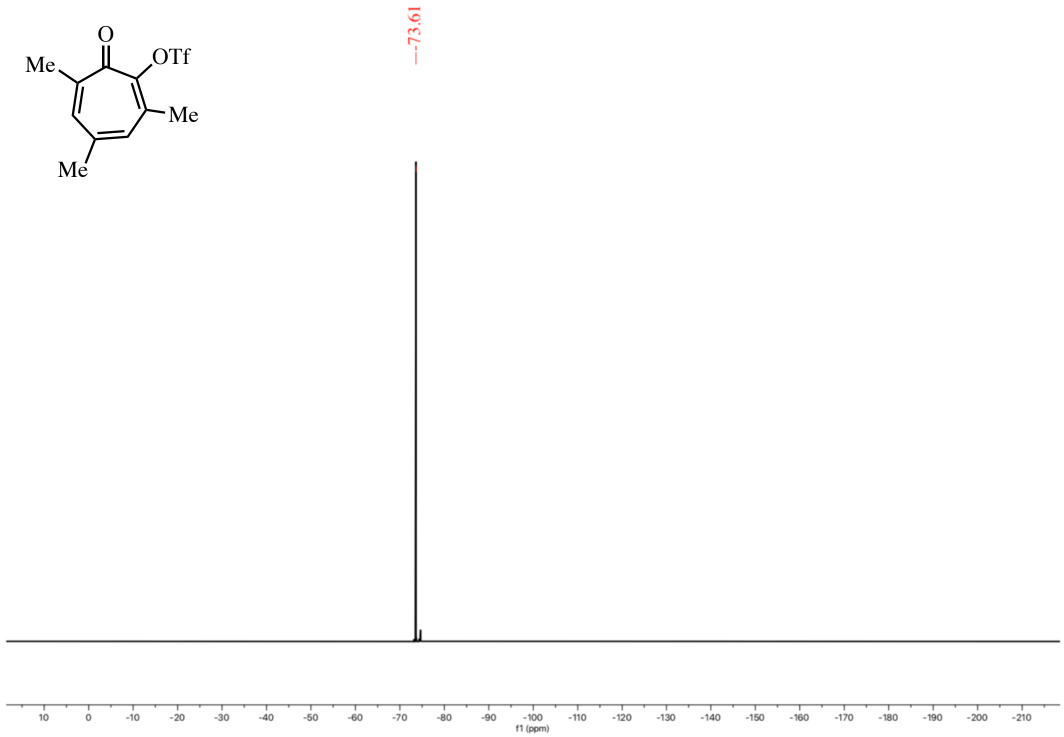

^{1}H-NMR (300 MHz) of G4Pd-L3

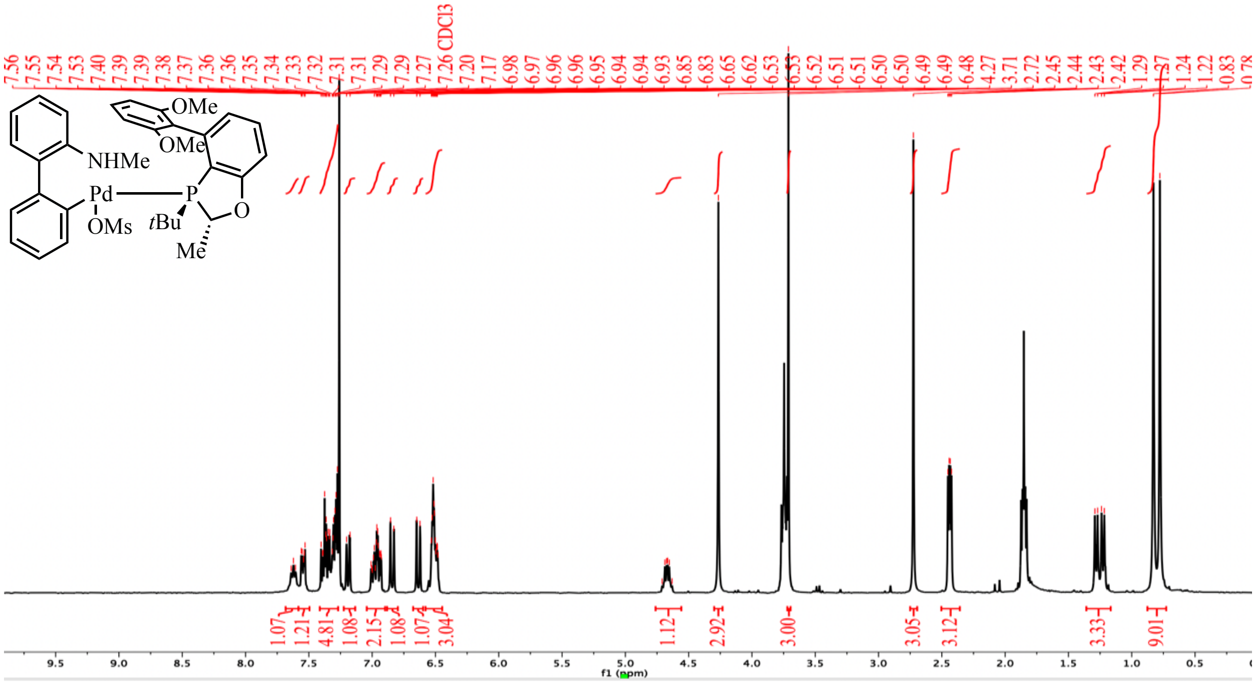

$^{13}C\{^{1}H\}$-NMR (75 MHz) of G4Pd-L3

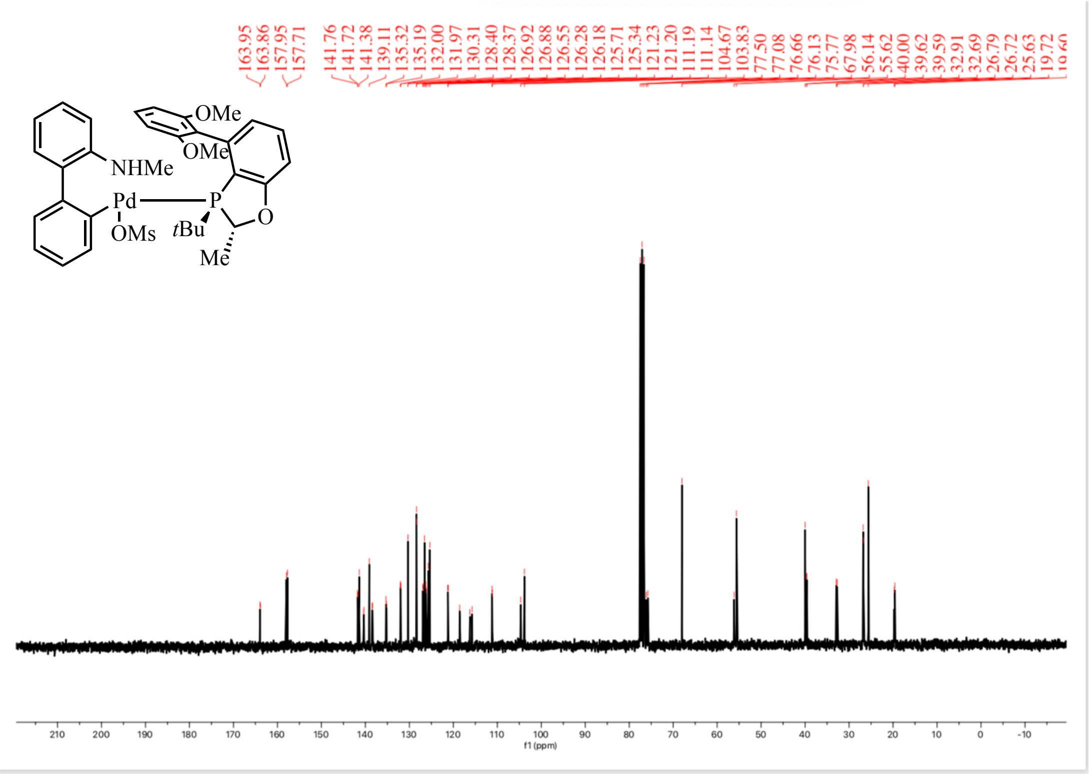

^{31}P-NMR (121 MHz) of G4Pd-L3

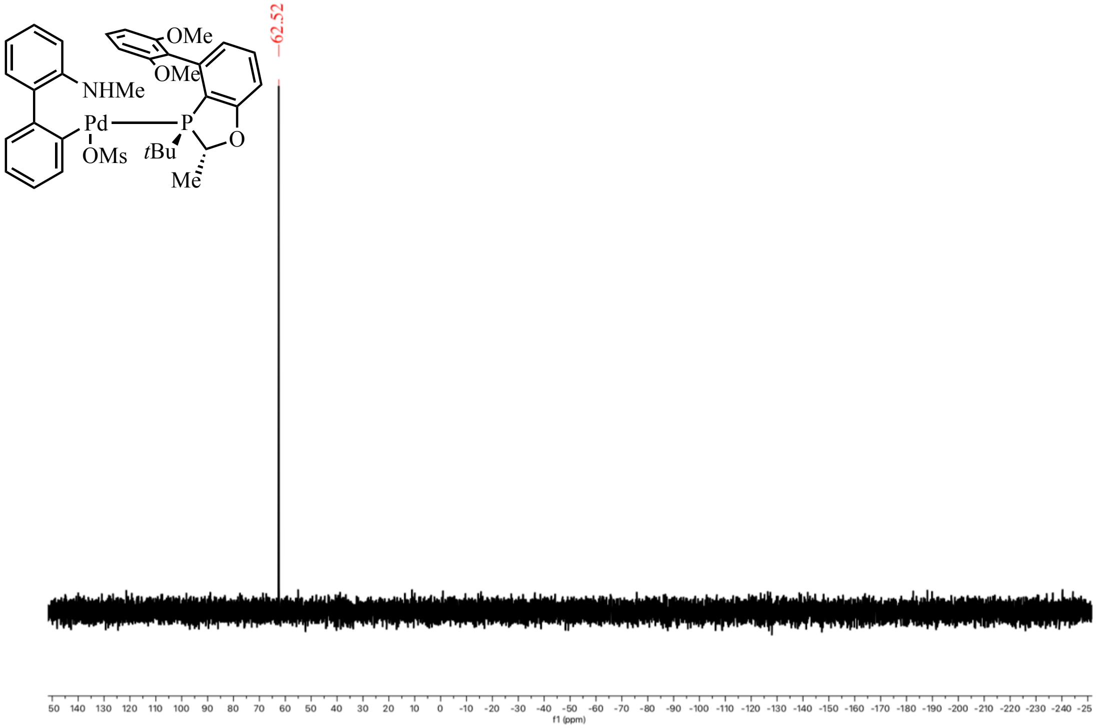

^{1}H-NMR (300 MHz) of 6a

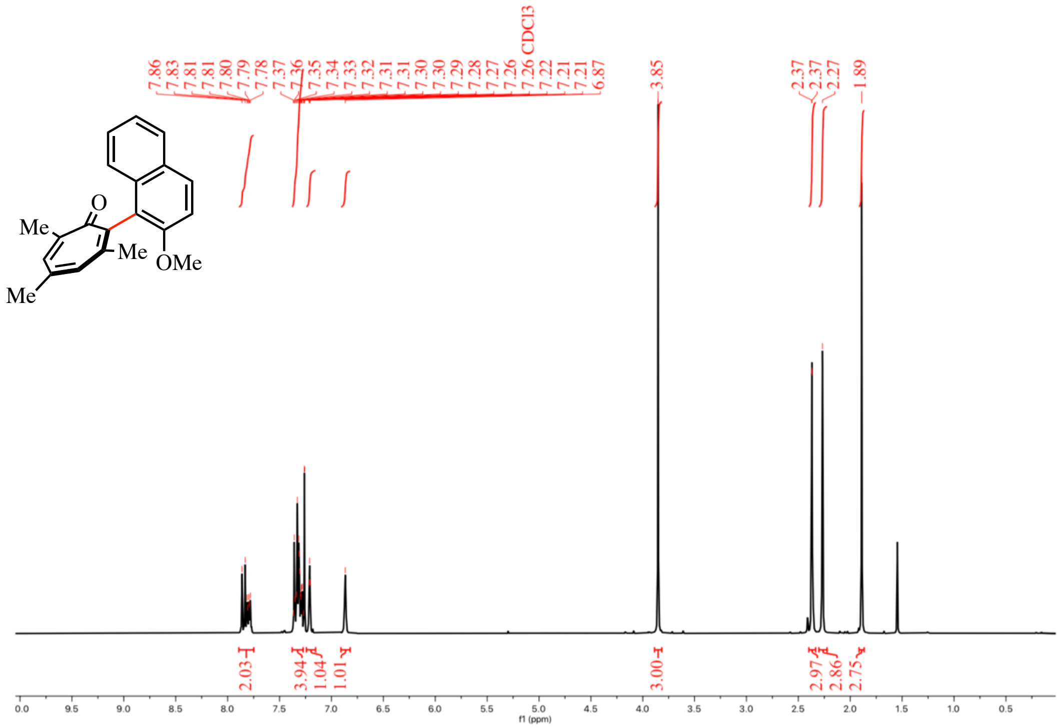

^{13}C{^{1}H}-NMR (75 MHz) of 6a

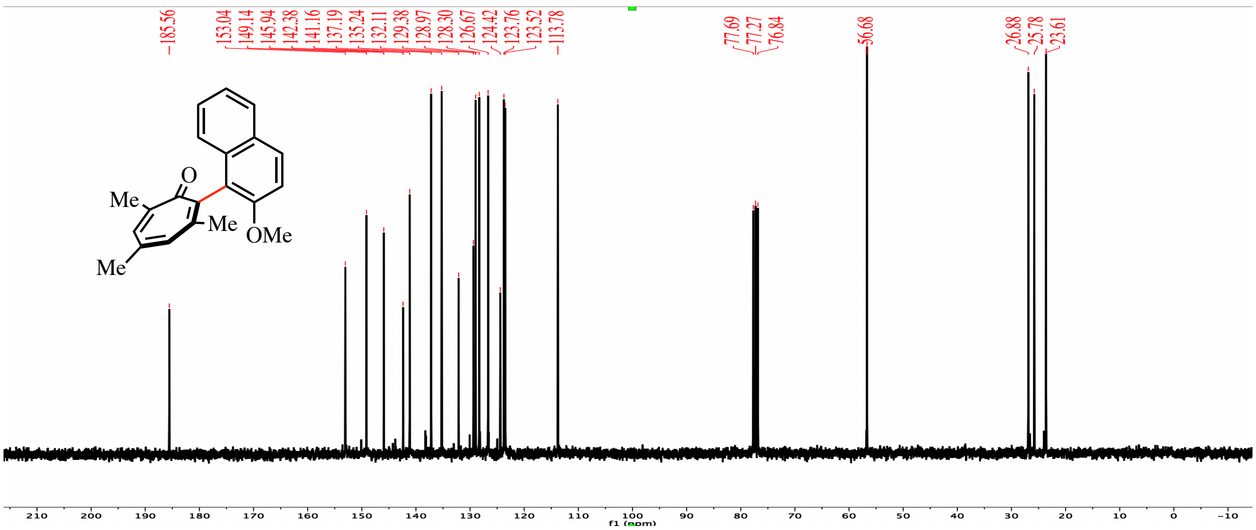

^{1}H-NMR (300 MHz) of 6b

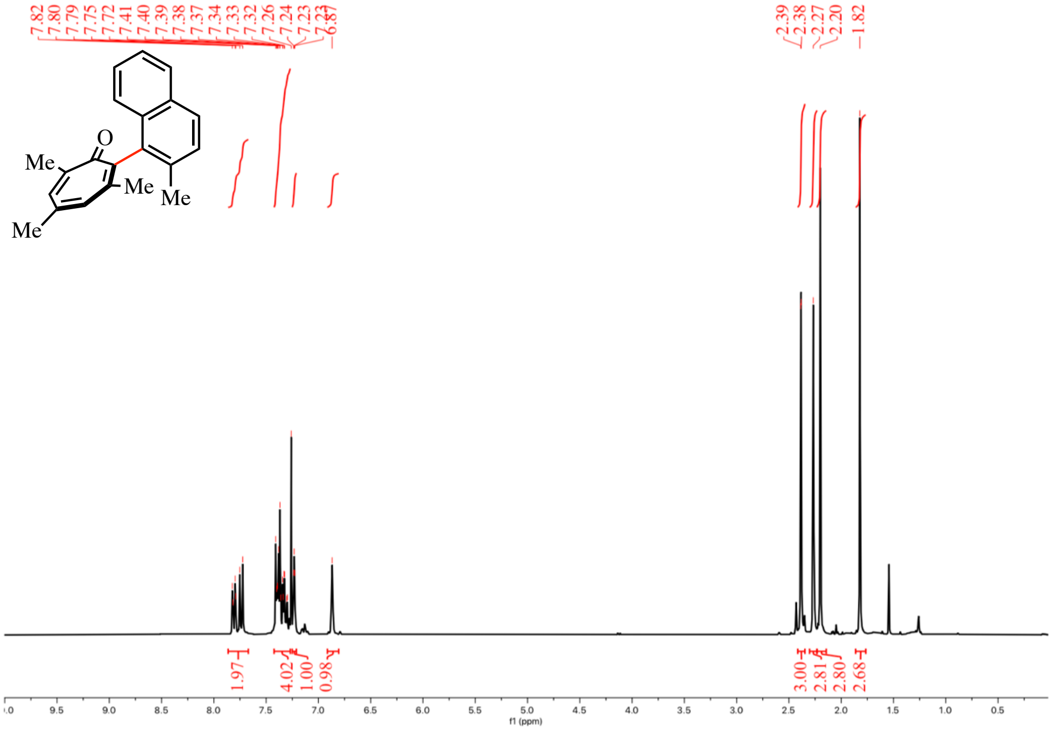

^{13}C{^{1}H}-NMR (75 MHz) of 6b

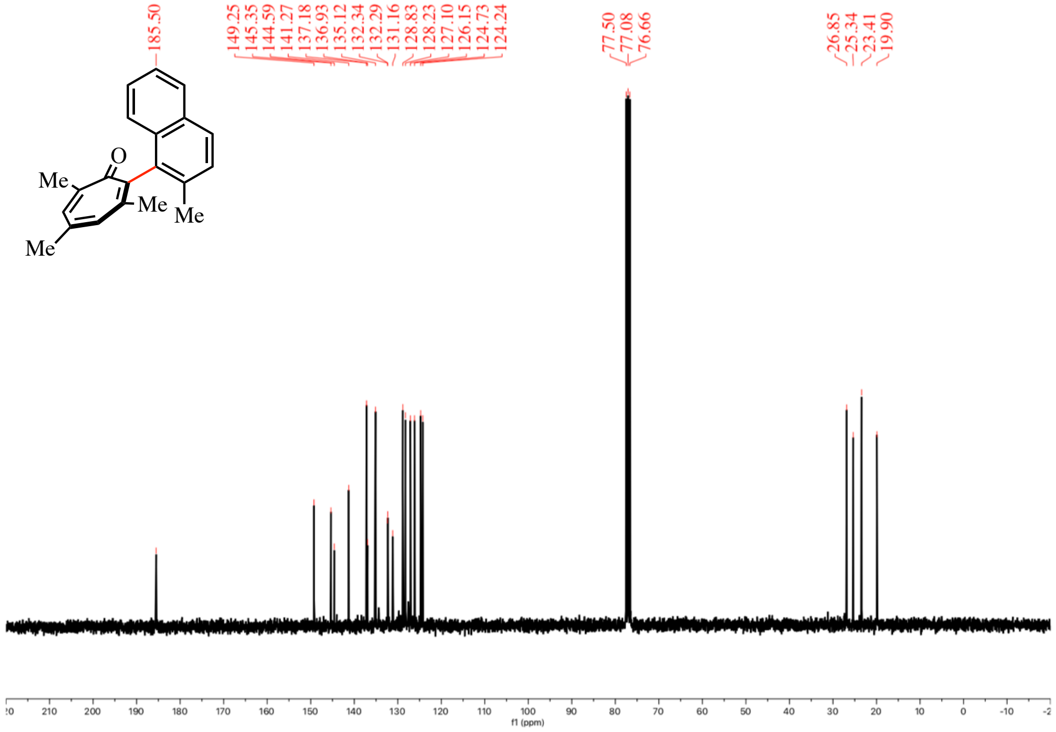

^{1}H-NMR (300 MHz) of 6f

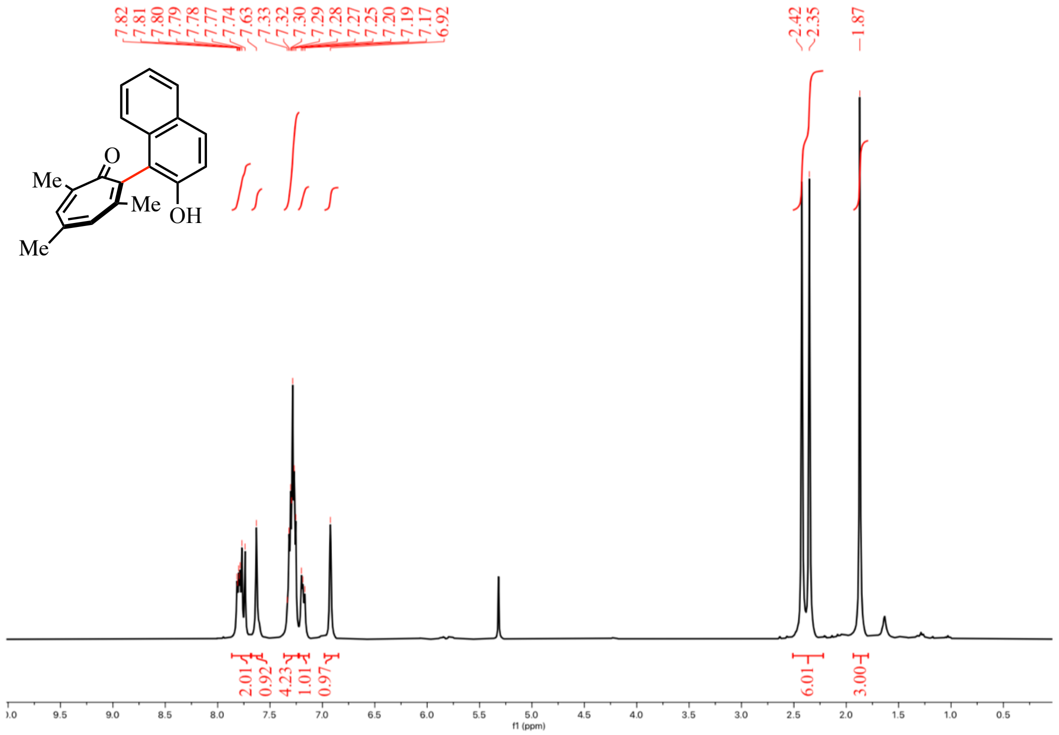

^{13}C{^{1}H}-NMR (75 MHz) of 6f

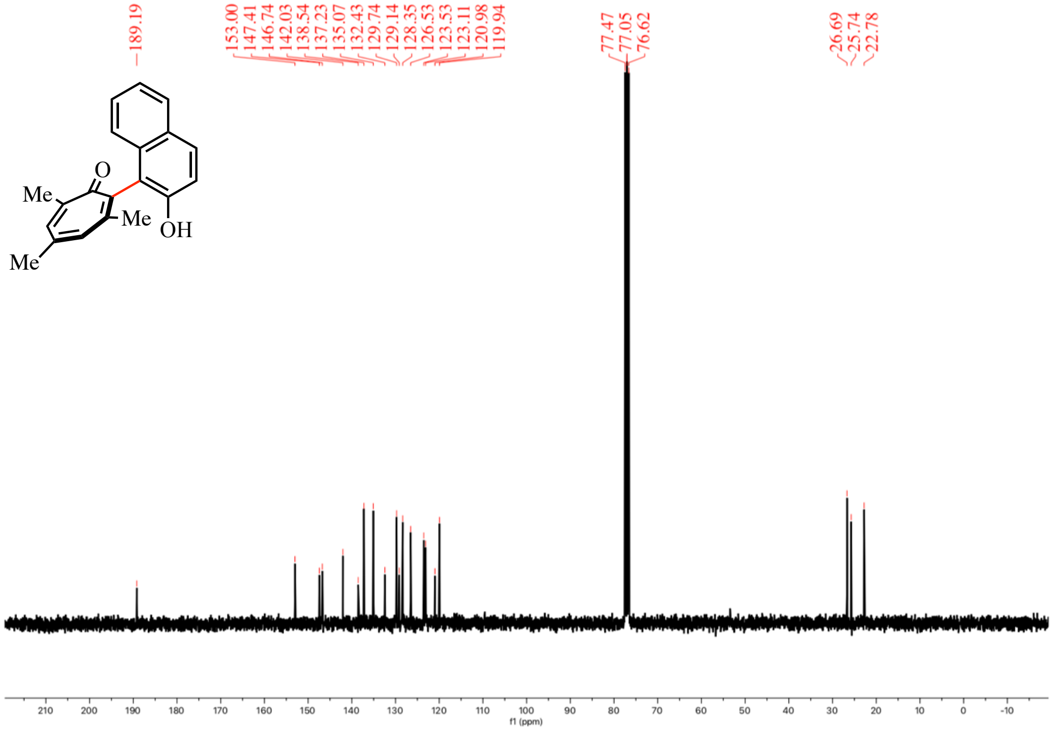

^{1}H-NMR (300 MHz) of 6d

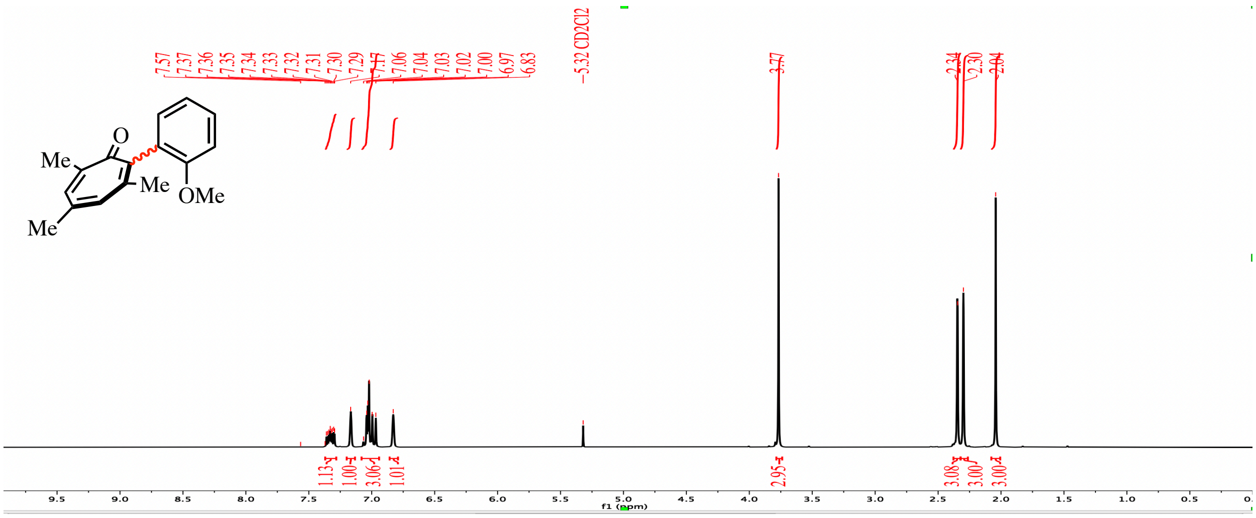

^{13}C{^{1}H}-NMR (75 MHz) of 6d

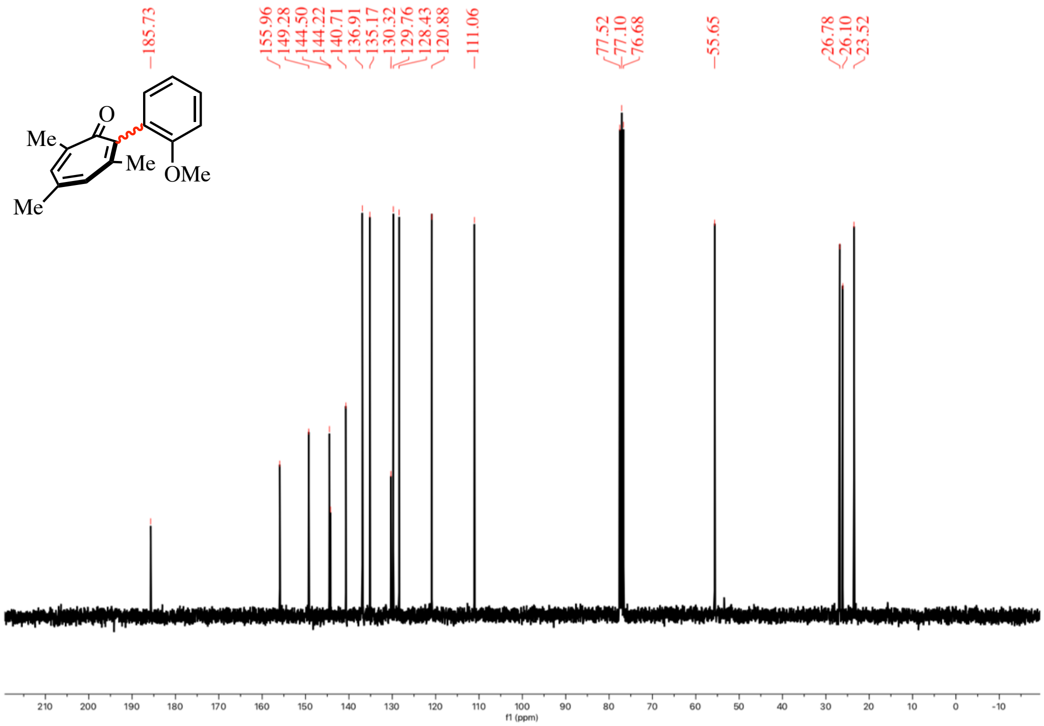

^{1}H-NMR (300 MHz) of 6e

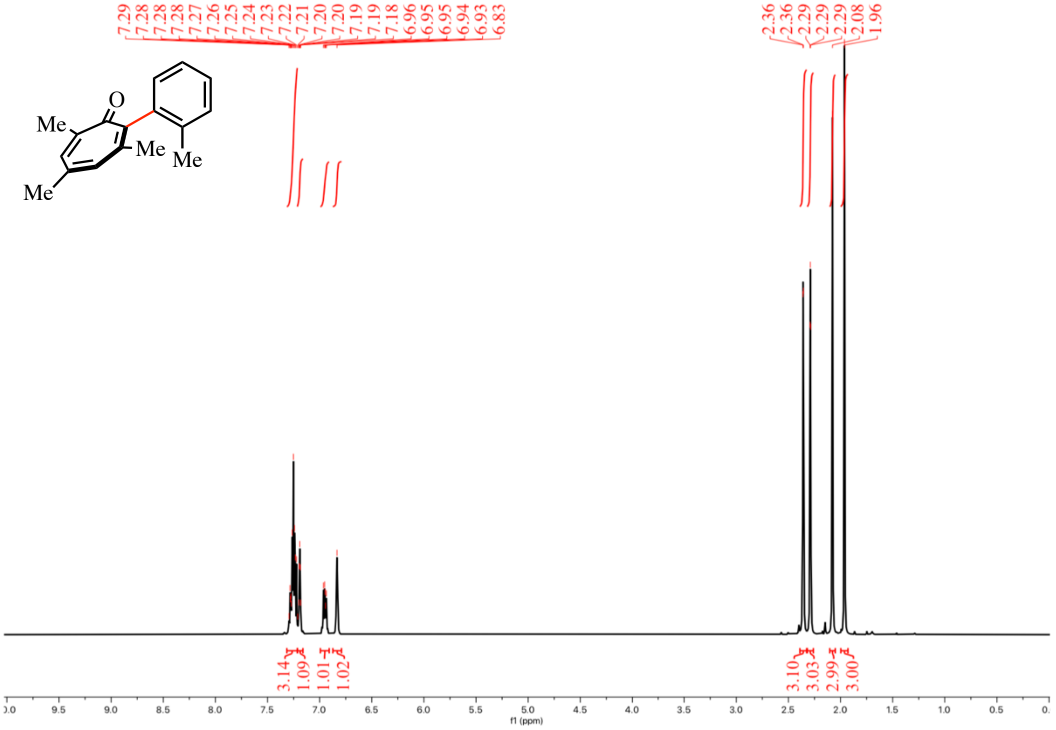

^{13}C{^{1}H}-NMR (75 MHz) of 6e

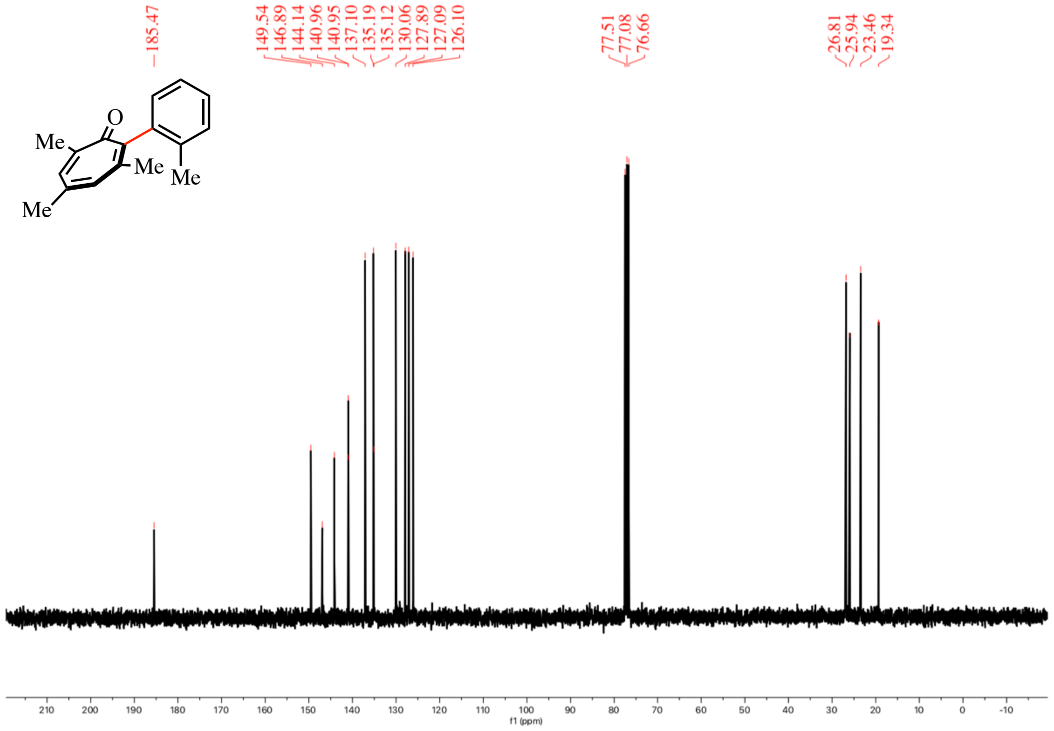

^{1}H-NMR (300 MHz) of 6c

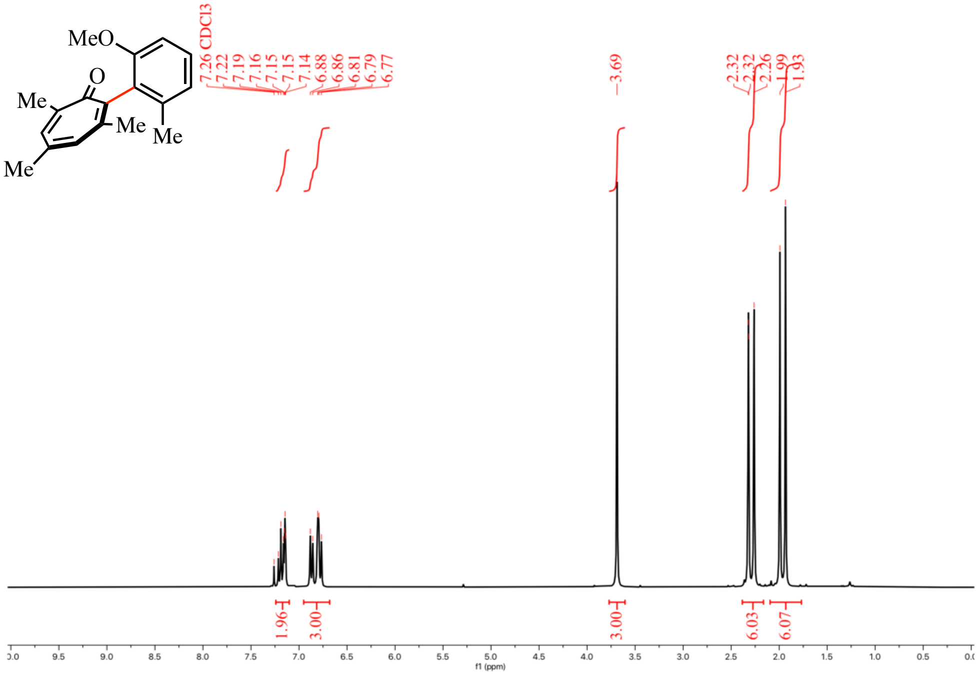

^{13}C{^{1}H}-NMR (75 MHz) of 6c

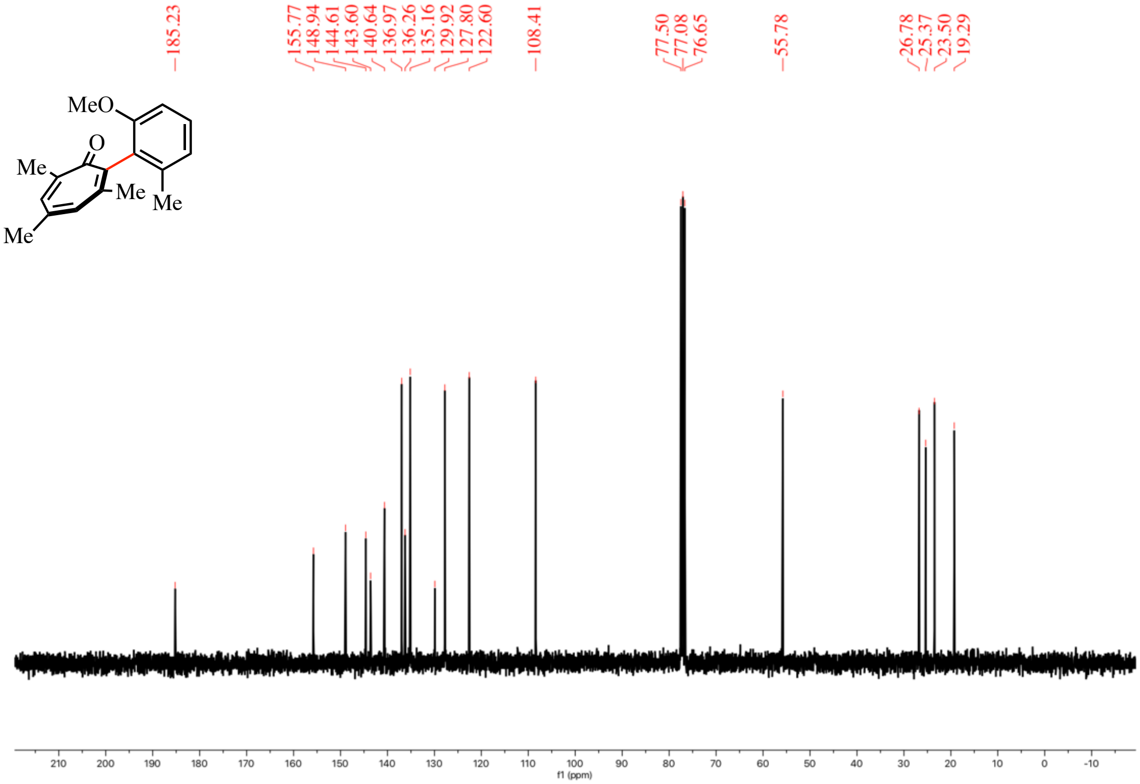

Racemization studies

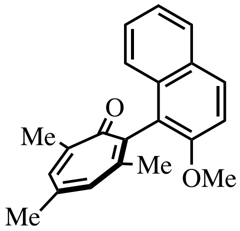

40.7 mg in 4 mL m-xylene heated, using oil bath, at 120 ºC

0 h	1 h	19 h	25 h	42 h	49 h
77.7 % ee	74.2% ee	34.7% ee	23.5% ee	9.1% ee	5.9% ee

30.5 mg in 4 mL m-xylene heated, using oil bath, at 120 ºC – obtained 32.2 kcal/mol

0 h	1 h	5 h	22 h	30 h	48 h
78.1% ee	74.1% ee	59.1% ee	16.5% ee	10.7% ee	6.1% ee

30 mg in 20 mL toluene heated, using oil bath, at 100 ºC

0 h	24 h	48 h	72 h	96 h	120 h
79.3% ee	73% ee	69.1% ee	68.9% ee	55.6% ee	47.7% ee

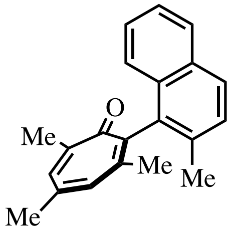

35.8 mg in 4 mL of m-xylene heated, using oil bath, at 120 ºC

0	1 h	5 h	22 h	30 h	48 h	53
72.4% ee	72.9	73.1	72.7	72.5	72.3	72.3

The fluctuations should be ignored. We can say it stayed the same.

32.9 mg in 4mL m-xylene heated, using oil bath, at 135 ºC

0	1 h	3 h	8 h	24 h	99 h	171 h
73.5	73.5	73.5	73.5	72.6	72.5	72.3

18.3 mg in 4 mL diphenyl ether heated, using oil bath, at 175 ºC

0	1 h	3 h	22 h	45 h	71 h	94 h	166 h
74% ee	74	74	74	72	70	69	63

Computational Rotational Barrier Measurements. All computational studies were carried out with Jaguar (Schrödinger Suite) in the gas phase at 298.15K at B3LYP-D3/6-31G+** theory and level. Compounds were first submitted to a geometry optimization, and then the optimized structures underwent a torsional scan using the software's relaxed coordinate scan feature. For consistency, each of these experiments started with an 80 ° dihedral, and the dihedral was incrementally changed (in 5 ° steps) past 360 ° to 420 ° (i.e., 60 °), in order to capture both transition states. The data from these studies are reported in the 'Relaxed Coordinate Scan' graphs below as 'change in gas phase energy'. These values were obtained by subtracting the ground state value and multiplying by 627.5 to convert from au to kcal/mol.

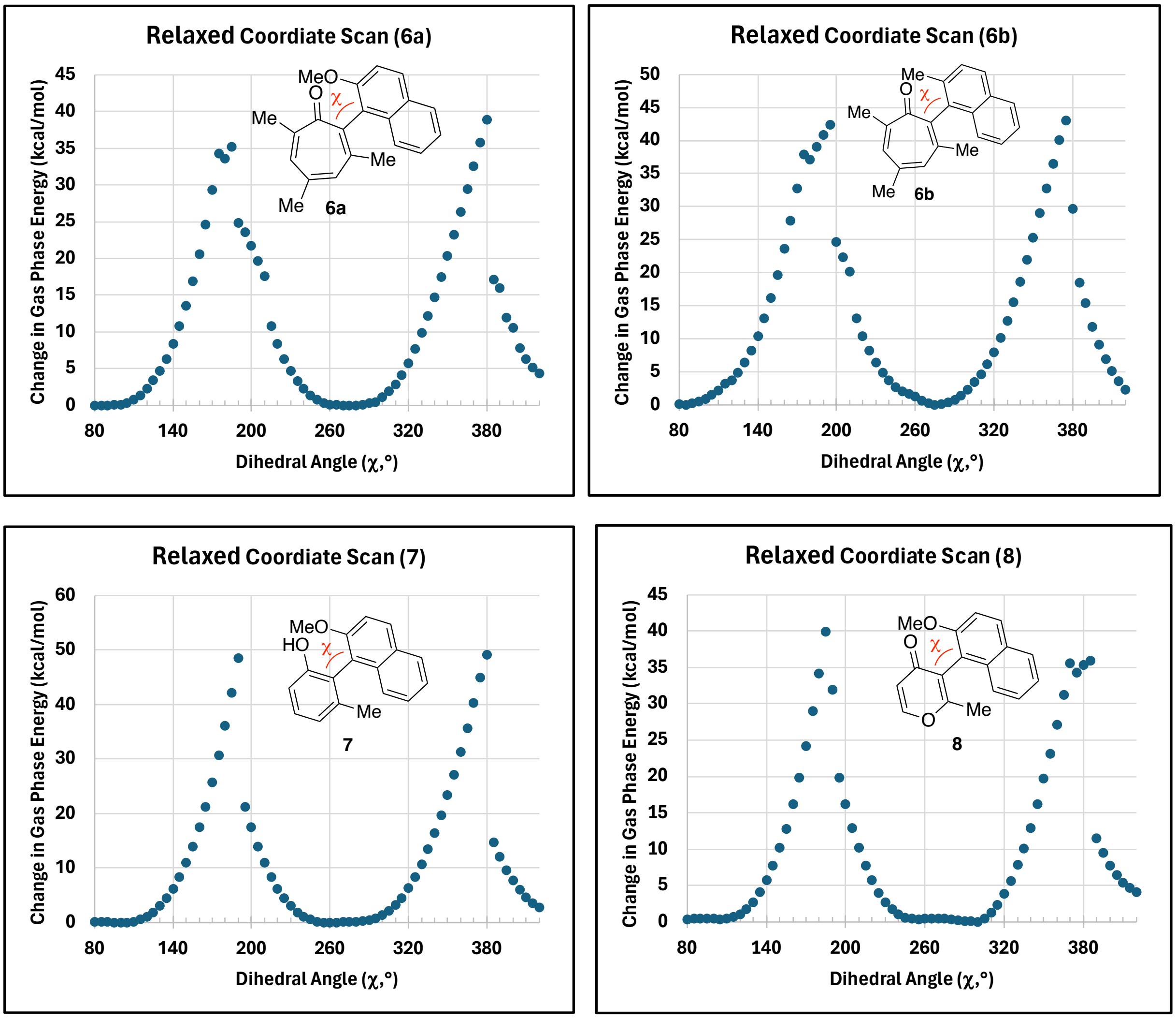

Figure SI 3. Plots obtained from relaxed coordinate scan focused on dihedral angle changes.

To obtain refined transition state energies, the maxima were refined into a transition state using the transition state search feature in Jaguar, with energy calculated with an associated vibrational frequency measurement (Table SI 1). Transition states were deemed adequate if they had a single negative frequency, and if an intrinsic reaction coordinate scan led towards the two ground-state axial chiral forms (Figure SI 4). Individual transition state energies ($\Delta G^{\ddagger}$) were determined in kcal/mol by subtracting optimized ground state structures, and then multiplying by 627.5 to convert from au to kcal/mol.

Predicted 'observed' ($\Delta G^{\ddagger}_{obs}$) were obtained by first identifying rate constants for each transition state using the Arrhenius equation:

$k_{trans} = (k_b T/h)e^{-\Delta G/kbT}$

T = 298.15 K, $k_B = 1.38 \times 10^{-23}$ m^2kg/s^2K, $h = 6.6 \times 10^{-34}$ m^2kg/s^2, $R = 1.99 \times 10^{-3}$ kcal/molK

Adding the pairs of rate constants (k_{trans}) for each compound provided k_{obs}, which can be added back into the Arrhenius equation to obtain $\Delta G^{\ddagger}_{obs}$:

$\Delta G^{\ddagger}_{obs} = -RT*\ln(k_B T/hk_{obs})$

k_{obs} can also be used to generate the half-life for enantiomerization using the following formula:

$t_{1/2} = \ln(2)/k_{obs}$

Data from these studies are included in the following tables:

Compound	**6a** (GS)	**6a** (TS1)	**6a** (TS2)
d (χ, °)	91.2	185.6	1.3
Gas Phase Energy (au)	-962.80543	-962.74892	-962.74874
Free Energy (au)	-962.49906	-962.44151	-962.44136
Lowest VF (cm-1)	18.6	-63.8	-74.4
2nd Lowest VF (cm-1)	33.0	45.3	37.9
$\Delta G^{\ddagger}$ (kcal/mol), 298.15K		36.1	36.2
K_{trans} (s-1)		2.3361E-14	1.9738E-14
K_{obs} (s-1)			4.31E-14
$t_{1/2, 298.15K}$ (y)			500,000
$\Delta G^{\ddagger}_{obs}$ (kcal/mol)			35.7

Compound	**6b** (GS)	**6b** (TS1)	**6b** (TS2)
d (χ, °)	83	193.9	7.5
Gas Phase Energy (au)	-887.59797	-887.52928	-887.52983
Free Energy (au)	-887.29517	-887.22579	-887.22578
Lowest VF (cm-1)	26.4	-82.2	-53.8
2nd Lowest VF (cm-1)	34.5	45.7	49.3
$\Delta G^{\ddagger}$ (kcal/mol), 298.15K		43.5	43.5
K_{trans} (s-1)		8.95E-20	8.95E-20
K_{obs} (s-1)			1.79E-19
$t_{1/2, 298.15K}$ (y)			100,000,000,000
$\Delta G^{\ddagger}_{obs}$ (kcal/mol)			43.1

Compound	**7** (GS)	**7** (TS1)	**7** (TS2)
d (χ, °)	105.6	182.3	17.5
Gas Phase Energy (au)	-846.08848	-846.02475	-846.02144
Free Energy (au)	-845.83918	-845.77375	-845.77022
Lowest VF (cm-1)	35.4	-26.1	-140
2nd Lowest VF (cm-1)	61.9	63.2	62.1
$\Delta G^{\ddagger}$ (kcal/mol), 298.15K		41.1	43.3
K_{trans} (s-1)		5.1123E-18	1.2539E-19
K_{obs} (s-1)			5.24E-18
$t_{1/2, 298.15K}$ (y)			4,000,000,000
$\Delta G^{\ddagger}_{obs}$ (kcal/mol)			41.1

Compound	**8** (GS)	**8** (TS1)	**8** (TS2)
d (χ, °)	105.2	191.9	25.7
Gas Phase Energy (au)	-881.97452	-881.92409	-881.92284
Free Energy (au)	-881.74933	-881.69705	-881.69577
Lowest VF (cm-1)	25.4	-40.3	-51.0
2nd Lowest VF (cm-1)	56.6	60.4	51.1
$\Delta G^{\ddagger}$ (kcal/mol), 298.15K		32.8	33.6
K_{trans} (s-1)		6.08E-12	1.58E-12
K_{obs} (s-1)			7.66E-12
$t_{1/2, 298.15K}$ (y)			3,000
$\Delta G^{\ddagger}_{obs}$ (kcal/mol)			32.7

Table SI 1. Tabulated data on ground state (GS) and transition states (TS) from computational modeling.

Figure SI 4. Plots from Intrinsic Reaction Coordinate Scans

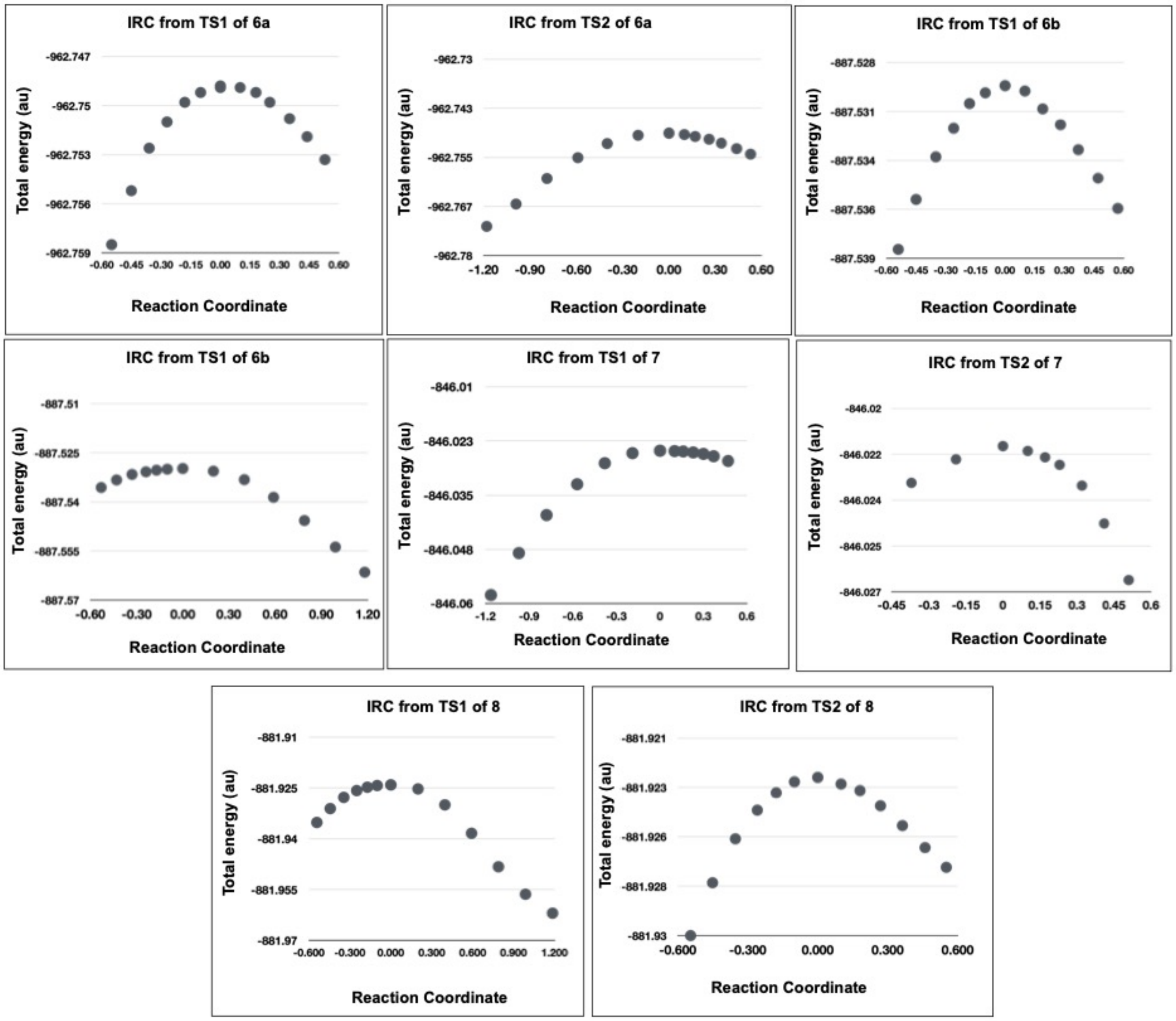

Nuclear Independent Chemical Shift Measurements. Various dihedral conformations of **6a** – generated from the aforementioned relaxed coordinate scan - were repositioned into the X/Y plane (ie, Z = 0) using a reference tropone structure and the superimposition feature in Maestro. For puckered structures, only the alkenes at position 2-3 and 6-7 were chosen to be superimposed. Dummy atoms were generated first by creating a centroid between all seven atoms in the tropone ring, and then generating a series by directly editing the xyz files (Cartesian coordinate files) and editing only on the Z axis, + or – a specific amount from the centroid. Initial studies on the ground-state (85°) evaluated every 0.2 Å from –2.0 Å to 2.0 Å. Subsequent studies on intermediate structures focused on a subset of these (+ and – 1.8 Å; 1.6 Å; 1.4 Å; 1.0 Å; 0 Å). Jaguar single point energy calculation were carried out at the B3PW91/6-311G+** theory and level, with the NMR spectra prediction tool selected during the experiment. Referring to the output file, total isotropic shielding calculated for dummy atoms provides -NICS(n), with n corresponding to distance from centroid, and the values specific to the ZZ shielding tensors provides -NICS(n)zz. Multiplying these values by -1 provides NICS(n) and $NICS(n)_{zz}$ as described in the following table:

Table SI 2. Tabulated data of NICS(n) and NICS(n)$_{zz}$ of tropone of **6a** at four different conformations. The dummy atoms used for obtaining date are shown in green in the structures.

Ground State (85 °)		
n (Å)	NICS(n)	NICS(n)zz
2	1.90	0.05
1.8	2.25	8.80
1.6	2.62	9.40
1.4	2.98	9.65
1.2	3.25	9.32
1	3.32	8.13
0.8	3.07	5.89
0.6	2.45	2.71
0.4	1.55	-0.88
0.2	0.65	-3.91
0	0.13	-5.35
-2	1.93	8.56
-1.8	2.28	9.26
-1.6	2.62	9.70
-1.4	2.89	9.97
-1.2	3.01	8.97
-1	2.87	7.33
-0.8	2.42	4.68
-0.6	1.69	1.28
-0.4	0.85	-2.18
-0.2	0.23	-4.69

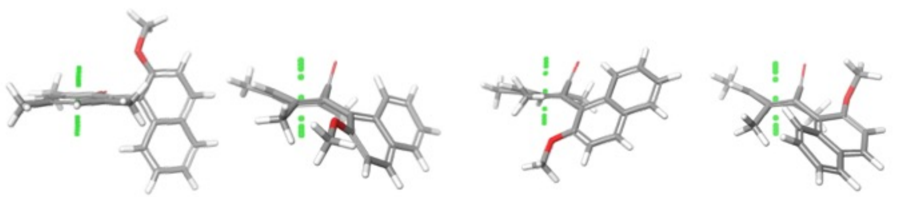

GS-6a TS1-6a Intermediate-6a TS2-6a

TS1 (185 °)		
n (Å)	NICS(n)	NICS(n)zz
0	3.26	-2.82
1	5.27	5.81
1.4	4.87	7.71
1.6	4.66	8.52
1.8	4.39	9.02
-1	5.23	5.10
-1.4	4.61	6.67
-1.6	4.11	6.66
-1.8	3.61	6.38

Intermediate (205 °)		
n (Å)	NICS(n)	NICS(n)zz
0	3.77	2.41
1	5.67	10.74
1.4	4.83	10.50
1.6	4.42	10.32
1.8	4.03	10.09
-1	5.36	9.94
-1.4	4.66	11.24
-1.6	4.04	10.81
-1.8	3.42	9.98

TS2 (385/25 °)		
n (Å)	NICS(n)	NICS(n)zz
0	2.30	-5.10
1	4.46	2.53
1.4	4.71	5.60
1.6	4.76	6.97
1.8	4.63	7.86
-1	4.59	3.02
-1.4	3.88	4.52
-1.6	3.36	4.61
-1.8	2.88	4.47
