# Supplementary material for: Synthetic, Computational, and Experimental Studies of a Class 3 Atropisomeric α‑Naphthyl Tropone
Source: J Org Chem. 2025 Aug 5;90(32):11501–9. doi: 10.1021/acs.joc.5c00992 (PMC12362610; doi:10.1021/acs.joc.5c00992)
Supplement: Supplementary file 2 [file jo5c00992_si_002.pdf]

Title:

VCD Absolute Configuration Determination Report

**GENERAL INFORMATION**	
**Customer**	CUNY Brooklyn
**Sales Order Number**	
**Sample code (BT ref.)**	**methoxy α-naphthyl tropone**
**Sample description (Customer ref.)**	**methoxy α-naphthyl tropone**
**VCD-spectrometer**	ChiralIR w/ Dual*PEM*
**Report prepared by: (name / signature as needed)**	Jordan Nafie
**Report validated and signed by**	Rina K Dukor
**Date**	February 13, 2024
**RESULTS**	
Absolute Configuration of **methoxy α-naphth tropone** is **(aS)**	Confidence Level: **100%**
**MEASUREMENT PARAMETERS**	
**Concentration**	9.7mg / 120uL
**Solvent**	$CDCl_3$
**Instrument Resolution**	4 cm^{-1}
**PEM setting**	1400 cm^{-1}
**Number of scans/Measurement time**	12 hours enantiomer and solvent
**Sample cell**	BaF_2
**Path length**	100 μm
**CALCULATION DETAILS**	
**Molecular Mechanics Force Field**	**MMFF94 (Compute VOA)**
**DFT Software version**	**Gaussian '09**
**Number of conformers used for Boltzmann sum**	**1 (cc-pVTZ / B3PW91)**
**Methodology and basis sets for DFT calculations**	**6-31G(d), cc-pVTZ / B3PW91 / CPCM ($CDCl_3$)**
**Enantiomer used for calculation**	**aS**
**Total calculated conformers**	**6**
**Number of low-energy conformations shown in report**	**1**

COMMENTS

The confidence level is a measure of the degree of congruence between a calculated and measured spectrum. If identical spectra are being compared the confidence level is 100%. The confidence level (CL) is not the likelihood that the assignment is correct. Rather it's a measure of quality or degree of agreement between calculated and measured spectra. With a CL of 100% for this molecule, the visual agreement between measured and calculated spectra is excellent – this is a very high confidence assignment. Four different DFT calculations were performed on this structure, two basis sets (6-31G(d) and cc-pVTZ) each with the functionals B3PW91 and B3LYP. All four calculations gave good results and the same answer for the stereochemistry. The combination of cc-pVTZ / B3PW91 had the best visual match and overlap values in CompareVOA. Despite the ~80% ee material, the VCD was quite robust as is common for atropisomeric compounds.

17546 Bee Line Hwy | Jupiter, Florida 33478, USA
ph. 561 625.0133 | fax. 561 625.0717 | www.biotools.us

Title:

VCD Absolute Configuration Determination Report

Structure of methoxy α-naphthyl tropone:

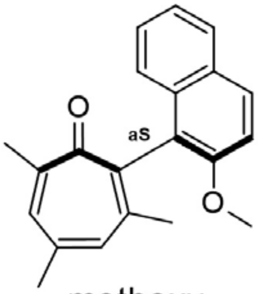

methoxy
α-naphthyl tropone

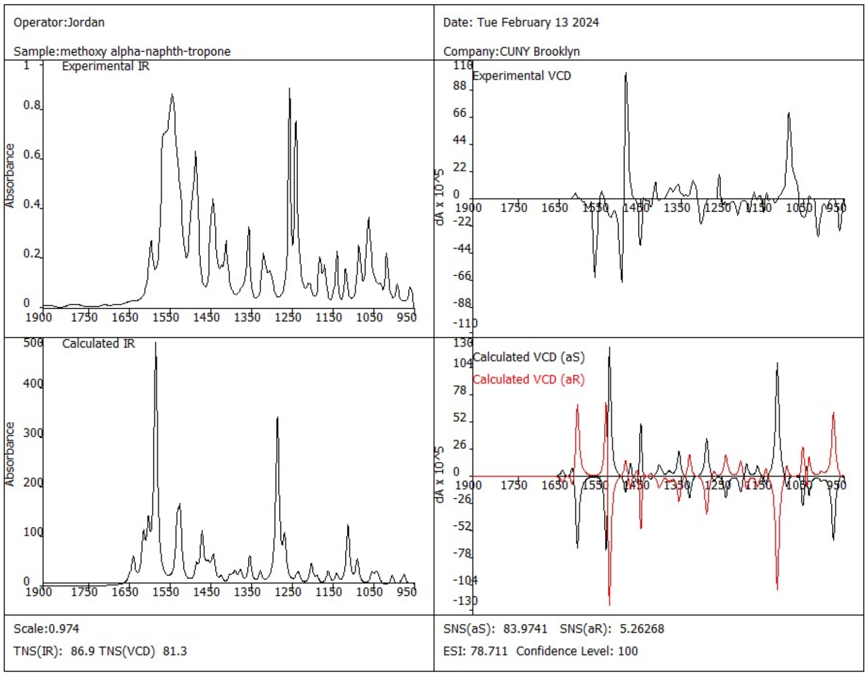

CompareVOA Results.
Please note that the calculated spectra are not scaled in this view.

17546 Bee Line Hwy | Jupiter, Florida 33478, USA
ph. 561 625.0133 | fax. 561 625.0717 | www.biotools.us

Table 1. Numerical comparison describing the similarity in the range of 950- 1900 cm^{-1} between the calculated IR and VCD spectra for the **(aS)** enantiomer at the cc-pVTZ / B3PW91 w/ CPCM (Chloroform) level and the observed IR and VCD spectra for **methoxy α-naphthyl tropone**.

Cal. (950-1900cm^{-1})	Numerical comparison	Observed **methoxy α-naphthyl tropone**
**(aS)**	scaling factor	0.974
	IR similarity (%)	86.9
	$^{a}\sum$ (%)	83.9741
	$^{b}\Delta$ (%)	78.711
	Confidence Level (%)	100

$^{a}\sum$: single VCD similarity, gives the similarity between the calculated and observed VCD spectra.
$^{b}\Delta$: enatiomeric similarity index, gives the difference between the values of ∑ for both enantiomers of a given diastereoisomer.

More information about CompareVOA can be found in the reference listed at the end of this report.

VCD Absolute Configuration Determination Report

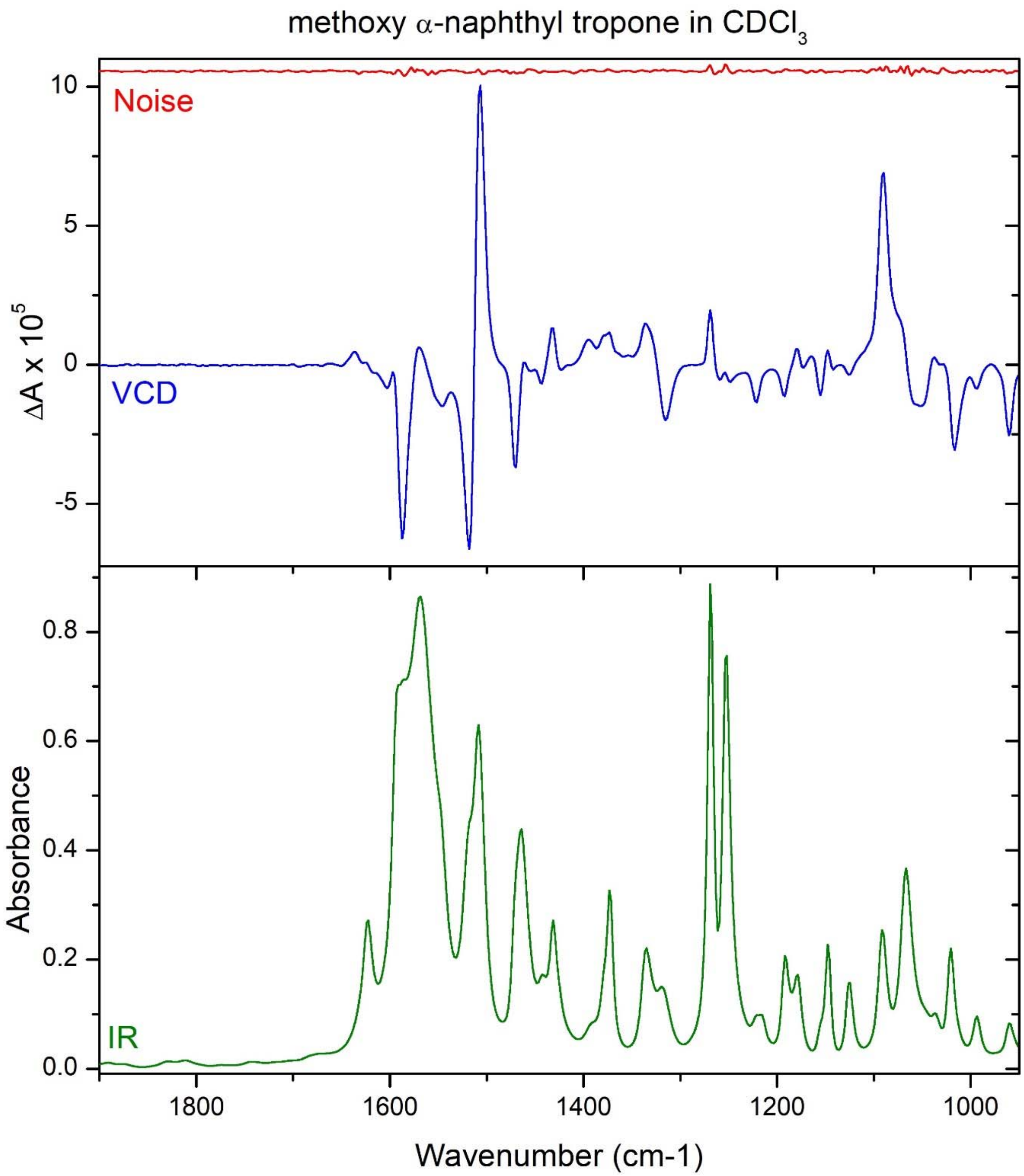

IR (lower frame) and VCD (upper frame) spectra of **methoxy α-naphthyl tropone** in $CDCl_3$; 100um path-length cell with BaF_2 windows; 12 h collection for enantiomer and solvent; instrument optimized at 1400 cm^{-1}. Solvent subtracted IR and VCD spectra are shown. Uppermost trace is the VCD noise spectrum.

Title:

VCD Absolute Configuration Determination Report

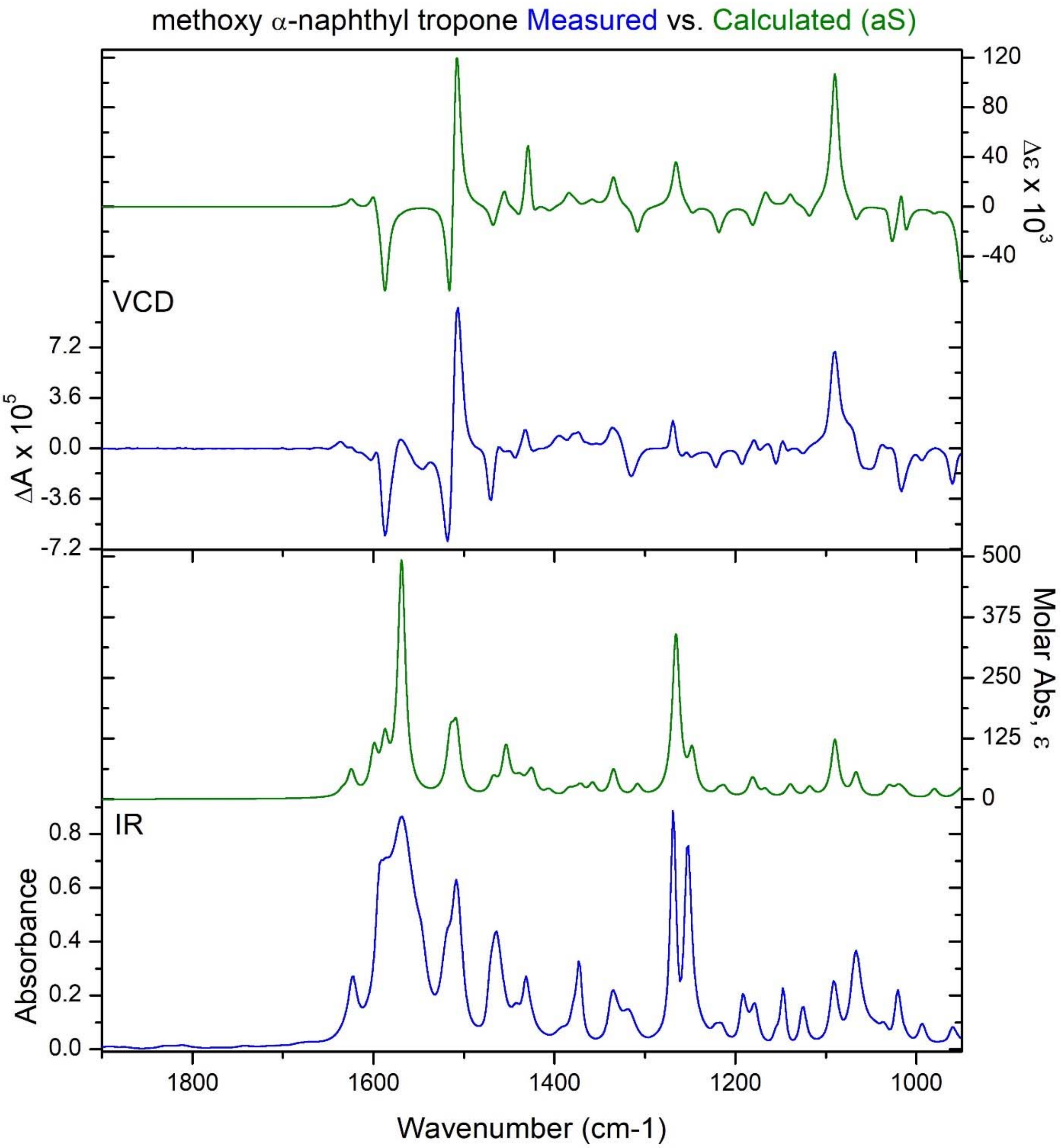

R (lower frame) and VCD (upper frame) spectra observed for **methoxy α-naphthyl tropone** (left axes) compared with Boltzmann-averaged spectra of the calculated conformations for the **(aS)** configuration, (right axes).

Single conformer used in DFT calculations: (aS) Configuration:

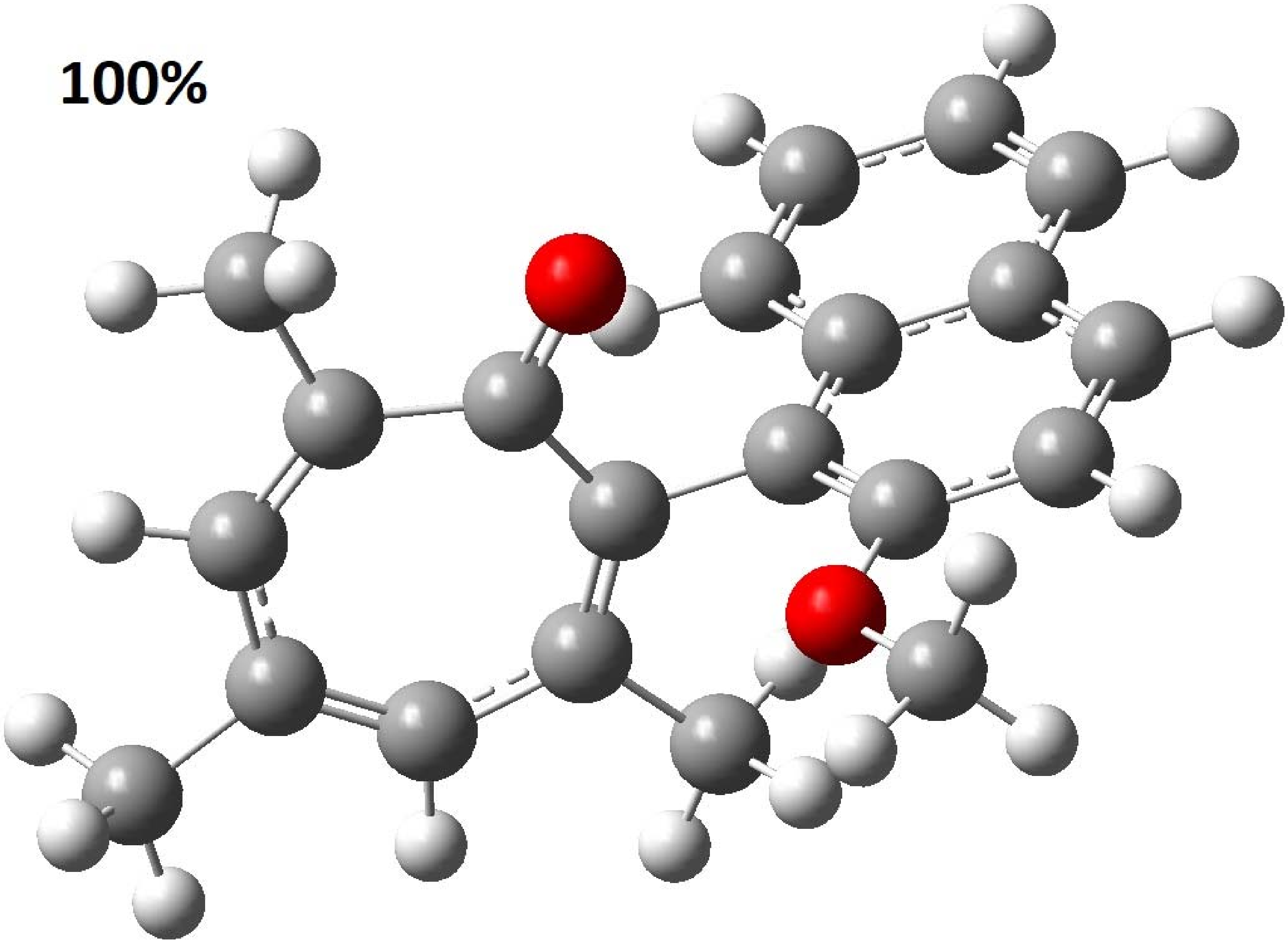

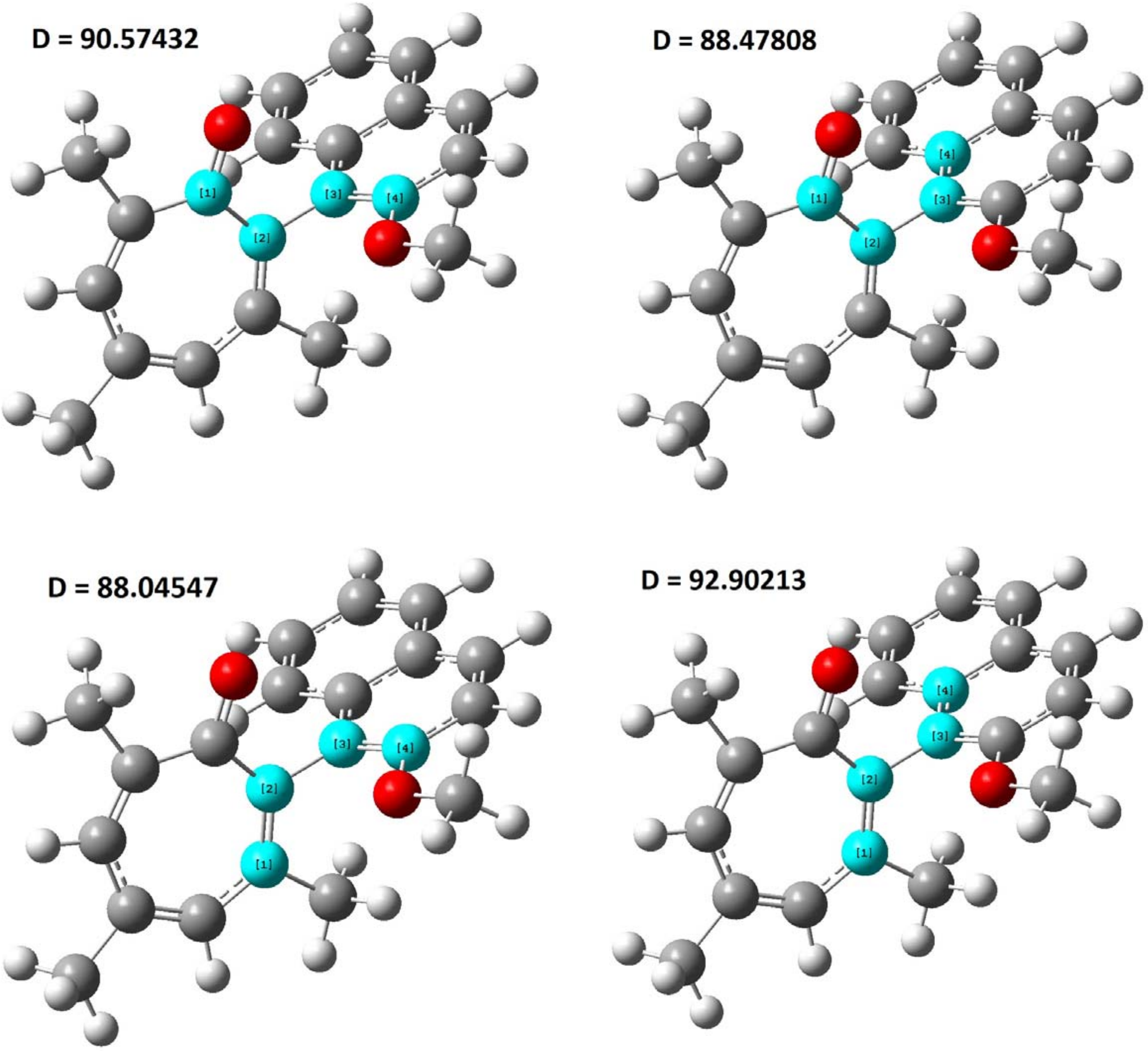

Dihedral angles of the chiral axis as measured using Gaussview (cc-pVTZ / B3PW91 opt).

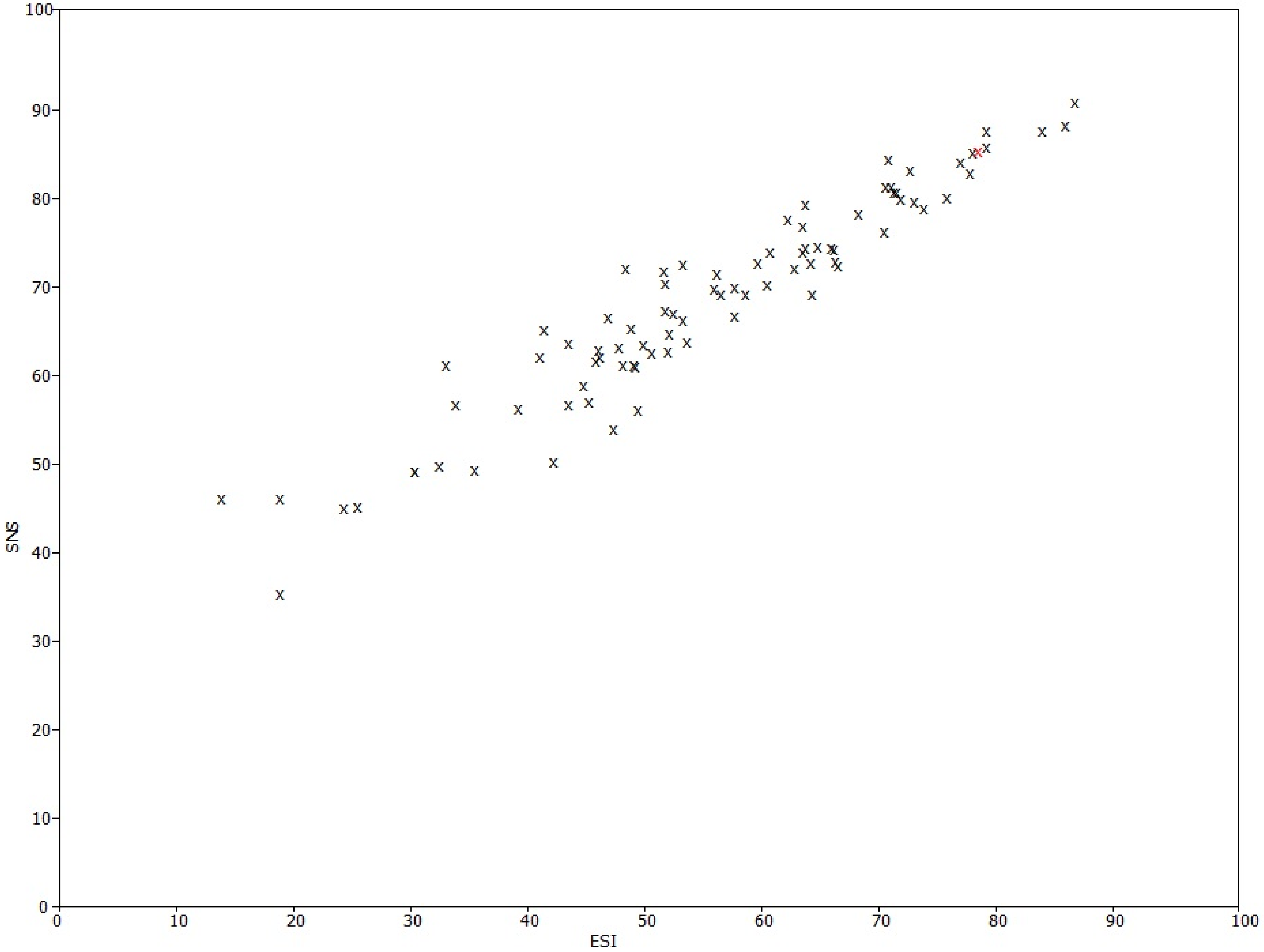

CompareVOA Plot of ESI (similarity of correct enantiomer minus incorrect enantiomer to calculated) vs SNS (overall similarity of correct enantiomer to calculated) for a library of correct assignments verified independently by X-Ray other method (Black X marks). Upper right corner are strongest assignments. Red X is **methoxy α-naphthyl tropone vs. (aS)**.

ChemPhysChem ***2011***, *12,* 1542 – 1549. (Reference available upon request).
